# RNA-Seq of Liver From Pigs Divergent in Feed Efficiency Highlights Shifts in Macronutrient Metabolism, Hepatic Growth and Immune Response

**DOI:** 10.3389/fgene.2019.00117

**Published:** 2019-02-19

**Authors:** Justyna Horodyska, Ruth M. Hamill, Henry Reyer, Nares Trakooljul, Peadar G. Lawlor, Ursula M. McCormack, Klaus Wimmers

**Affiliations:** ^1^Teagasc, Food Research Centre, Ashtown, Ireland; ^2^Leibniz Institute for Farm Animal Biology (FBN), Institute for Genome Biology, Dummerstorf, Germany; ^3^Teagasc, Pig Production Department, AGRIC, Moorepark, Fermoy, Co. Cork, Ireland; ^4^Faculty of Agricultural and Environmental Sciences, University Rostock, Rostock, Germany

**Keywords:** FE, RFI, residual feed intake, gene expression, transcriptomics

## Abstract

Liver is a metabolically complex organ that influences nutrient partitioning and potentially modulates the efficiency of converting energy acquired from macronutrients ingestion into a muscle and/or adipose tissue (referred to as feed efficiency, FE). The objective of this study was to sequence the hepatic tissue transcriptome of closely related but differently feed efficient pigs (*n* = 16) and identify relevant biological processes that underpin the differences in liver phenotype between FE groups. Liver weight did not significantly differ between the FE groups, however, blood parameters showed that total protein, glucose, cholesterol and percentage of lymphocytes were significantly greater in high-FE pigs. Ontology analysis revealed carbohydrate, lipid and protein metabolism to be significantly enriched with differentially expressed genes. In particular, high-FE pigs exhibited gene expression patterns suggesting improved absorption of carbohydrates and cholesterol as well as enhanced reverse cholesterol transport. Furthermore, the inferred decrease in bile acid synthesis in high-FE pigs may contribute to the observed greater levels of serum glucose, which can be then delivered to cells and utilized for growth and maintenance. Gene ontology analysis also suggested that livers of more efficient pigs may be characterized by higher protein turnover and increased epithelial cell differentiation, whereby an enhanced quantity of invariant natural killer T-cells and viability of natural killer cells could induce a quicker and more effective hepatic response to inflammatory stimuli. Our findings suggest that this prompt hepatic response to inflammation in high-FE group may contribute to the more efficient utilization of nutrients for growth in these animals.

## Introduction

Liver is a central organ for systemic metabolism (Rui, 2014; Shimizu et al., 2015) and plays an important role in modulating the efficiency of converting energy acquired from macronutrients into muscle and/or adipose tissue affecting feed efficiency (FE). Energy from ingested macronutrients can be stored by the liver in the form of glycogen, which during food deprivation is broken down into glucose and delivered to the bloodstream (Sherwin, 1980; Zhang et al., 2014). Liver can also convert energy from a dietary source to triacylglycerol and export it by very low density lipoproteins (VLDL) either to muscle for use there or to adipose tissue for storage (Gruffat et al., 1996). Moreover liver is a key organ for synthesis of cholesterol, a vital constituent of cell membrane, and lipoproteins that function as cholesterol transporting particles (Charlton-Menys and Durrington, 2008). Low density lipoproteins (LDL) deliver cholesterol to peripheral organs, whilst high density lipoproteins (HDL) transport excess cholesterol from these tissues back to the liver (Feingold and Grunfeld, 2000), which is then utilized, e.g., for synthesis of bile acids that enable intestinal absorption of dietary fats (Boyer, 2013).

Being continually subjected to antigens entering from the gut via blood supply, liver also exhibits immunological properties (Gao, 2016; Peng et al., 2016). The lymphocyte population of the liver is primarily represented by macrophages, natural killer and natural killer T cells that are involved in innate immune defense and regulation of liver regeneration (Racanelli and Rehermann, 2006; Gao, 2016). Fueling immune response is an energetically expensive process resulting in less nutrients available for growth (Patience et al., 2015). This alteration in prioritizing nutrients toward stimulating immune response would be suspected to negatively impact animal’s FE. Nevertheless, it has been postulated that high-FE animals have a more efficient immune response to fight off inflammation and therefore more energy available for growth and muscle deposition (Paradis et al., 2015).

Transcriptomic approach is a relevant tool in developing a deeper understanding of the physiological processes in liver which may be related to FE. To date only a few studies have analyzed the transcriptome of liver from FE-divergent pigs wherein shifts in biological processes that were observed to be differentially regulated in association with FE included cell proliferation, vitamin A metabolism, protein synthesis and catabolism, lipid and carbohydrate metabolism, reverse cholesterol transport, integrin signaling, as well as oxidative stress, inflammation and immune responses (Zhao et al., 2016; Gondret et al., 2017; Reyer et al., 2017; Ramayo-Caldas et al., 2018). Additionally, another study investigated mitochondrial proteomics of liver from pigs divergent in FE and as a result identified a shift in mitochondrial protein profile (Grubbs et al., 2013). Although these studies have offered insights on the regulation of FE via liver physiology, the biological processes governing the differences in FE are not fully elucidated and further research is needed. Hence the purpose of the present study was to characterize phenotypes relevant to liver physiology and to perform RNA sequencing of liver tissue in pigs divergent for FE to gain deeper insights into differences in hepatic transcriptome architecture and its relationship to liver function in more efficient pigs.

## Materials and Methods

### Ethics Statement

The care, slaughter and sample collection of the animals fulfilled the national regulations of animal research and commercial slaughtering and were approved by the Teagasc Animal Ethics Committee for the care and use of animals.

### Animals and Experimental Design

A total of 138 Maxgro (Hermitage Genetics) × (Landrace × Large White) pigs were used in this study. Housing, diets, and selection on FE were as previously described (Horodyska et al., 2018a). Briefly, residual feed intake (RFI, a measure of FE defined as the difference between actual feed intake and predicted feed requirements) was calculated after day 120 of age as the residual from a least squares regression model of average daily feed intake on average daily gain, metabolic live weight, gender and also all relevant two-way interactions, and the effects of back-fat and muscle depth using the PROC REG procedure in SAS (version 9.4; SAS Inst. Inc., Cary, NC, United States). According to RFI values, pigs were assigned within litter and gender as high (H) and low (L) RFI. The minimum and maximum RFI values (g/day) for the 138 pigs were -329 and 494, respectively. A total of 40 pigs (20 extremes from LRFI (high-FE) group – 10 males and 10 females, and 20 extremes from HRFI (low-FE) group – 10 males and 10 females), with an average body weight of 99 kg, were sampled considering the relatedness of pigs. The mean RFI (g/day) of the LRFI and HRFI pigs was -100.2 (SD: 97.9) and 150.7 (SD: 163.3), respectively.

### Phenotypic Measurements

For each animal (*n* = 40), liver weights were recorded and blood samples were collected in vacuette tubes (ROI: Labstock, Dublin, Ireland; AT: Sarstedt, Nürnbrecht, Germany) during slaughter. For biochemical analysis, upon allowing the blood to clot at room temperature, the samples were centrifuged at 1,500 × *g* for 10 min and the serum was collected and stored at -80°C until analyzed. Creatinine, creatine kinase, total protein, blood urea nitrogen, triglycerides, glucose and cholesterol were analyzed with a calibrated ABS Pentra 400 clinical chemistry analyzer (Horiba, ABX, North Hampton, United Kingdom). In order to determine analyzer accuracy, every fifth sample was run in duplicate. For hematological analysis, blood was treated with EDTA to prevent clotting. It was then subjected to analysis, within 4 h of sample collection, whereby white blood cells, lymphocytes, monocytes, granulocytes, red blood cells, red blood cell distribution width, hemoglobin, hematocrit, mean corpuscular volume, mean corpuscular hemoglobin, platelets and mean platelet volume were measured with a Beckman Coulter Ac T Diff analyzer (Beckman Coulter Ltd., High Wycombe, United Kingdom). The PROC MIXED procedure in the SAS was used to evaluate associations between FE and liver weight as well as biochemical and hematological parameters in the Maxgro × (Landrace × Large White) pigs (*n* = 40). The model included RFI group as a fixed effect, slaughter day as a random effect, and the absolute values of RFI as a weight statement. Additionally for liver weight, final live body weight was incorporated in the model as a covariate. Moreover, correlations between RFI and hematological and biochemical parameters were determined using the PROC CORR procedure in the SAS system (version 9.4; SAS Inst. Inc., Cary, NC, United States).

### RNA Sequencing of Liver Samples, Data Processing, and Ontology Analysis

Samples of the right liver lobe (*Lobus Spigelii*) tissue were collected and snap frozen in liquid nitrogen within 10 min *post-mortem* followed by storage at -80°C until RNA isolation. Of these 40 liver tissues from RFI-divergent pigs, 16 samples from four sets of full siblings were selected and each set consisted of 2 males – 1 LRFI (high-FE) and 1 HRFI (low-FE) and 2 females 1 LRFI (high-FE) and 1 HRFI (low-FE) so that 8 LRFI (high-FE) pigs – 4 males and 4 females and 8 HRFI pigs (low-FE) – 4 males and 4 females were analyzed. Total RNA of these liver samples was isolated with Tri-Reagent (Sigma-Alrich, Taufkirchen, Germany), and subjected to DNase treatment and a column-based purification (Nucleospin RNA II kit, Macherey-Nagel, Düren, Germany). Total RNA was used as input for the library preparation according to the TruSeq Stranded mRNA protocol (Illumina, San Diego, CA, United States). Subsequently, sequencing was performed on an Illumina HiSeq2500 generating paired-end reads. Reads were mapped to the reference (Ensembl release 84) using TopHat (2.1.0) (Kim et al., 2013) and read counts were assigned to the gene features employing HTSeq 0.6.1 (Anders et al., 2015). The assessment of the differentially expressed genes included RFI groups and slaughter dates as fixed effects and was performed using the Wald test implemented in DESeq2 (3.4.0)^1^. To integrate gene expression data, the list of DE genes (*P* < 0.01) and corresponding fold changes were passed to Ingenuity Pathway Analysis (IPA; Ingenuity^®^Systems)^2^ and significantly enriched bio-functions and canonical pathways (*P* < 0.01) were extracted. They were considered significantly activated and inhibited at an absolute z-score greater than 2. In addition, potential important interaction networks enriched with DE genes were generated using the Ingenuity^®^ Knowledge Base.

### Validation of RNA-Seq Through Quantitative Real-Time PCR (qPCR)

Following cDNA synthesis from 1 μg of total RNA and in presence of random primers (Promega, Mannheim, Germany), oligo (dT) primer and Superscript^®^III reverse transcriptase (Invitrogen Corp., San Diego, CA, United States), qPCR were carried out on a LightCycler 96 system (Roche Mannheim, Germany). Gene-specific primers (Table 1) were designed using the Primer-BLAST software^3^ and the BLAST search tool database^4^. PCR reactions were carried out in a final volume of 12 μl consisting of 2 μl cDNA, 6 μl SYBR Green I Master (Roche), 0.6 μl (10 μM) of each forward and reverse primer, and 2.8 μl qPCR grade water (Roche). After an initial denaturation at 95°C for 5 min, 45 cycles of amplification followed (95°C for 10 s, 60°C for 15 s and 72°C for 25 s). Melting curve analysis was performed at the end of the amplification to verify the specificity of all amplification reactions. *RPL32* expression values were used to normalize qPCR results. Subsequently, qPCR data was analyzed in a mixed model including RFI group as a fixed effect and slaughter date as a random effect (lme4; R). The correlation between RNA-seq and qPCR data were assessed in R considering a significance threshold of *P* < 0.05.

**Table 1:** Forward and reverse primers and amplicon length used for qPCR analysis.

Gene	NCBI accession no.	Forward	Reverse	Product size (bp)
*KIT*	NM_001044525.1	TTCTCGTGTCCAATGCTGATG	TCGGTGCCTGGACAGAAATAC	166
*PON3*	NM_001044604.1	CAATGGGATCACAGTCTCATCAG	TGCCCAAATATCTCCCGTATC	178
*SAA3*^∗^	NM_001044552.1	CTCAAGGAAGCTGGTCAAGG	GGACATTCTCTCTGGCATCG	178
*RPL32*	NM_001001636.1	AGCCCAAGATCGTCAAAAAG	TGTTGCTCCCATAACCAATG	165

^∗^Olsen et al. (2013).

## Results

### Phenotypic Measurements

Liver weight did not significantly differ between the FE groups (high-FE = 1.62 kg ± 0.04 kg and low-FE = 1.67 kg ± 0.04 kg). The effect of divergence in FE on biochemical and hematological parameters are shown in Table 2. Biochemical analysis of serum revealed that total protein, glucose and cholesterol were significantly (*P* < 0.05) higher in high-FE pigs compared to low-FE pigs. Creatinine, creatine kinase, blood urea nitrogen and triglycerides did not differ significantly between the groups. Hematological analysis exposed significantly (*P* < 0.05) reduced number of white blood cells but increased percentage of lymphocytes in high-FE pigs. The number of platelets and mean platelet volume was significantly (*P* < 0.001 and *P* < 0.05, respectively) lower in high-FE pigs. Remaining hematological parameters were not significantly altered by FE group. Spearman correlations between phenotypic parameters of pigs divergent in FE are depicted in Table 3. A number of significant correlations at a *P* < 0.001 were observed between phenotypes. The strongest linear relationships were noted between total protein and cholesterol (*r* = 0.781), glucose and cholesterol (*r* = 0.737), red blood cells and hemoglobin (*r* = 0.726), creatinine and cholesterol (*r* = 0.723), creatinine and total protein (*r* = 0.704), total protein and triglycerides (*r* = 0.629), total protein and glucose (*r* = 0.608), as well as creatinine and glucose (*r* = 0.601). This was followed by moderate linear relationships between triglycerides and creatinine (*r* = 0.566), cholesterol (*r* = 0.563) and blood urea nitrogen (*r* = 0.560).

**Table 2:** Effect of divergence in feed efficiency (FE) on biochemical and hematological parameters.

	Measurement	High-FE^a^	Low-FE^a^	SE	*P*-value
Biochemistry	Creatinine (μmol/L)	117.7	98.27	11.0	0.085
	Creatine kinase (μmol/L)	89.83	87.56	16.8	0.893
	Total protein (g/L)	61.03	48.21	6.26	**0.048**
	Blood urea nitrogen (mg/dL)	14.56	9.444	3.54	0.157
	Triglycerides (mmol/L)	0.620	0.549	0.07	0.351
	Glucose (mmol/L)	4.892	3.968	0.37	**0.016**
	Cholesterol (mmol/L)	2.329	1.811	0.25	**0.045**
Hematology	White blood cells (×10^3^ cells/μl)	22.66	27.30	1.84	**0.016**
	Lymphocytes (%)	51.94	42.93	3.43	**0.013**
	Monocytes (%)	7.226	6.466	1.04	0.469
	Granulocytes (%)	42.06	38.77	5.60	0.561
	Lymphocyte number (×10^3^ cells/μl)	11.51	11.24	1.08	0.809
	Monocyte number (×10^3^ cells/μl)	1.430	1.648	0.24	0.371
	Granulocyte number (×10^3^ cells/μl)	9.990	10.30	1.73	0.859
	Red blood cells (×10^6^ cells/μl)	6.386	6.772	0.35	0.274
	Hemoglobin (g/dL)	11.20	11.74	0.62	0.388
	Hematocrit (%)	0.352	0.353	0.01	0.870
	Mean corpuscular volume (fL)	52.69	52.57	0.70	0.864
	Mean corpuscular hemoglobin (%)	17.37	17.31	0.26	0.809
	Mean corpuscular hemoglobin conc (pg)	32.35	32.52	0.45	0.705
	Red cell distribution width (fL)	19.24	20.27	0.84	0.229
	Platelets (10^6^ cells/μl)	178.3	272.2	29.9	**0.003**
	Mean platelet volume (fL)	7.778	8.937	0.54	**0.039**

^a^*Least square means for each parameter. *P* < 0.05 are highlighted in bold*.

**Table 3:** Correlations between hematological and biochemical parameters.

	C	CK	TP	BUN	Tg	Glu	Chol	WBC	Lc	Mc	Gc	LcN	McN	GcN	RBC	Hg	Hc	MCV	MCH	MCHC	RCDW	P
CK	-0.070																					
	0.674																					
TP	0.704	-0.084																				
	<**0**.**001**	0.613																				
BUN	0.380	0.058	0.418																			
	**0**.**017**	0.727	**0**.**008**																			
Tg	0.566	0.032	0.629	0.560																		
	<**0**.**001**	0.846	<**0**.**001**	<**0**.**001**																		
Glu	0.601	-0.003	0.608	0.363	0.191																	
	<**0**.**001**	0.985	<**0**.**001**	**0**.**023**	0.245																	
Chol	0.723	-0.075	0.781	0.439	0.563	0.737																
	<**0**.**001**	0.651	<**0**.**001**	**0**.**005**	<**0**.**001**	<**0**.**001**																
WBC	-0.083	-0.025	-0.173	-0.365	-0.237	-0.121	-0.048															
	0.614	0.881	0.292	**0**.**023**	0.147	0.463	0.774															
Lc	0.188	-0.085	0.290	0.317	0.110	0.378	0.295	-0.476														
	0.253	0.606	0.074	0.050	0.506	**0**.**018**	0.068	**0**.**002**														
Mc	0.292	0.009	0.440	0.235	0.410	0.173	0.264	-0.539	0.577													
	0.071	0.959	**0**.**005**	0.150	**0**.**010**	0.292	0.105	<**0**.**001**	<**0**.**001**													
Gc	-0.158	0.033	-0.062	-0.009	-0.061	-0.176	-0.257	0.085	-0.434	-0.421												
	0.336	0.842	0.709	0.955	0.714	0.285	0.114	0.605	**0**.**006**	**0**.**008**												
LcN	0.131	-0.110	0.174	0.087	-0.063	0.377	0.307	0.214	0.690	0.180	-0.362											
	0.428	0.503	0.290	0.598	0.703	**0**.**018**	0.057	0.192	<**0**.**001**	0.274	**0**.**024**											
McN	0.247	-0.021	0.429	0.101	0.306	0.186	0.295	-0.264	0.476	0.902	-0.525	0.331										
	0.129	0.897	**0**.**006**	0.542	0.059	0.258	0.068	0.104	**0**.**002**	<**0**.**001**	**0**.**001**	**0**.**040**										
GcN	-0.154	-0.030	-0.014	-0.088	-0.059	-0.174	-0.184	0.323	-0.406	-0.392	0.930	-0.129	-0.388									
	0.350	0.856	0.932	0.596	0.722	0.290	0.261	**0**.**045**	**0**.**010**	**0**.**014**	<**0**.**001**	0.432	**0**.**015**									
RBC	0.397	-0.054	0.461	0.079	0.213	0.287	0.466	0.134	0.105	-0.017	0.001	0.289	0.103	0.091								
	**0**.**013**	0.746	**0**.**003**	0.632	0.193	0.077	**0**.**003**	0.415	0.527	0.918	0.994	0.075	0.532	0.580								
Hg	0.254	-0.016	0.220	-0.065	0.062	0.199	0.345	0.125	0.028	-0.148	0.011	0.214	-0.101	0.074	0.726							
	0.119	0.921	0.177	0.692	0.708	0.225	**0**.**032**	0.447	0.863	0.367	0.946	0.191	0.540	0.656	<**0**.**001**							
Hc	0.355	0.025	0.317	-0.010	0.161	0.274	0.445	0.240	0.015	-0.046	-0.028	0.250	0.014	0.062	0.656	0.742						
	**0**.**026**	0.882	**0**.**049**	0.954	0.326	0.091	**0**.**005**	0.141	0.927	0.780	0.866	0.125	0.933	0.707	<**0**.**001**	<**0**.**001**						
MCV	-0.173	0.201	-0.328	-0.259	-0.346	-0.030	-0.148	0.254	-0.316	-0.310	0.146	-0.137	-0.299	0.151	-0.304	0.140	0.320					
	0.293	0.220	**0**.**041**	0.111	**0**.**031**	0.857	0.369	0.119	0.050	0.055	0.375	0.407	0.065	0.360	0.060	0.397	**0**.**047**					
MCH	-0.146	0.323	-0.159	-0.229	-0.129	0.047	-0.012	0.220	-0.345	-0.290	0.178	-0.161	-0.250	0.170	-0.208	0.159	0.354	0.852				
	0.375	**0**.**045**	0.333	0.161	0.434	0.774	0.944	0.179	**0**.**032**	0.073	0.279	0.328	0.125	0.301	0.203	0.333	**0**.**027**	<**0**.**001**				
MCHC	0.146	0.163	0.309	-0.100	0.149	0.303	0.336	**0**.**019**	-0.147	-0.104	0.060	-0.082	-0.029	0.039	0.135	0.321	0.427	0.288	0.614			
	0.376	0.320	0.056	0.546	0.364	0.061	**0**.**037**	0.908	0.373	0.529	0.719	0.621	0.862	0.815	0.413	**0**.**046**	**0**.**007**	0.076	<**0**.**001**			
RCDW	-0.144	-0.066	-0.155	0.101	0.007	-0.282	-0.187	0.267	-0.020	-0.097	-0.123	0.127	-0.025	-0.083	0.164	-0.025	-0.123	-0.525	-0.488	-0.371		
	0.383	0.689	0.345	0.541	0.964	0.082	0.253	0.100	0.905	0.555	0.455	0.442	0.882	0.614	0.318	0.882	0.456	**0**.**001**	**0**.**002**	**0**.**020**		
P	-0.175	0.080	-0.189	0.106	0.009	-0.067	-0.077	0.193	-0.188	-0.259	-0.117	-0.041	-0.176	-0.086	-0.036	-0.047	-0.041	0.105	0.113	-0.013	0.025	
	0.287	0.628	0.249	0.521	0.957	0.687	0.641	0.240	0.253	0.112	0.479	0.803	0.283	0.601	0.827	0.776	0.803	0.524	0.492	0.937	0.878	
MPV	-0.131	-0.042	-0.186	-0.210	-0.166	-0.010	0.066	0.375	-0.409	-0.468	**0**.**024**	-0.174	-0.461	0.023	0.036	0.304	0.343	0.487	0.418	0.172	-0.060	0.334
	0.425	0.798	0.256	0.200	0.313	0.953	0.688	**0**.**019**	**0**.**010**	**0**.**003**	0.884	0.291	**0**.**003**	0.892	0.829	0.060	0.033	**0**.**002**	**0**.**008**	0.295	0.715	**0.038**

*Correlation coefficient is presented in the upper row and a P-value is shown in the bottom row. *P* < 0.05 are highlighted in bold*.

*Abbreviations: C: Creatinine (μmol/L), CK: Creatine kinase (μmol/L), TP: Total protein (g/L), BUN: Blood urea nitrogen (mg/dL), Tg: Triglycerides (mmol/L), Glu: Glucose (mmol/L), Chol: Cholesterol (mmol/L), WBC: White blood cells (x 103 cells/μl), Lc: Lymphocytes (%), LcN: Lymphocyte number (x 103 cells/μl), Mc: Monocytes (%), McN: Monocyte number (x 103 cells/μl), Gc: Granulocytes (%), GcN: Granulocyte number (x 103 cells/μl), RBC: Red blood cells (106 cells/μL), RCDW: Red cell distribution width (fL), Hg: Hemoglobin (g/dL), Hc: Hematocrit (%), MCV: Mean corpuscular volume (fL), MCH: Mean corpuscular hemoglobin (%), MCHC: Mean corpuscular hemoglobin concentration (pg), P: Platelets (106 cells /μL), MPV: Mean platelet volume (fL)*.

### Differentially Expressed Genes and Ontological Interpretation

**Figure 1:**
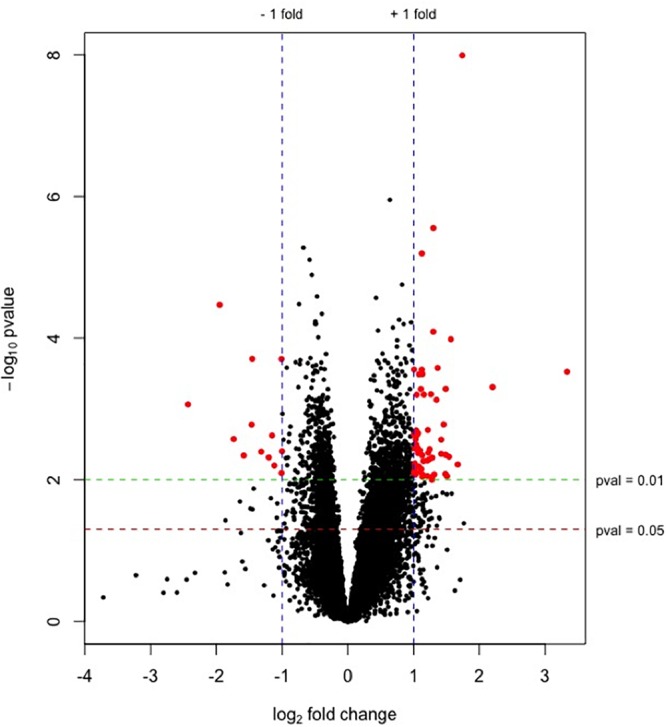
Plot of –log_10_(*p*-values) versus log_2_ fold changes for genes expressed in liver from high-FE pigs. The green and red lines indicate *P* = 0.01 and 0.05, respectively. Blue lines represent the threshold of genes with a log2 fold change ≥|1| (fold change ≥ |2|). Significantly differentially expressed genes (*P* < 0.01, log2 fold change ≥|1| [fold change ≥|2|]) are highlighted by red dots. Up-regulated genes in high-FE pigs are indicated by positive fold changes.

**Figure 2:**
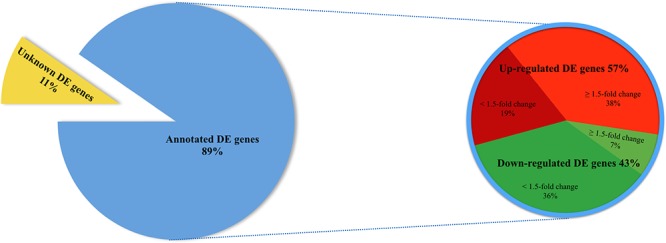
Pie chart depicting a total of 922 differentially expressed (DE) genes (*P* < 0.01) in liver of FE-divergent pigs and a percentage of annotated up- and down-regulated genes in high-FE pigs. The most up-regulated gene was *PON3* (fold change = 10.08) and the most down-regulated gene was *CCNT2* (fold change = -5.40) in high FE pigs.

**Figure 3:**
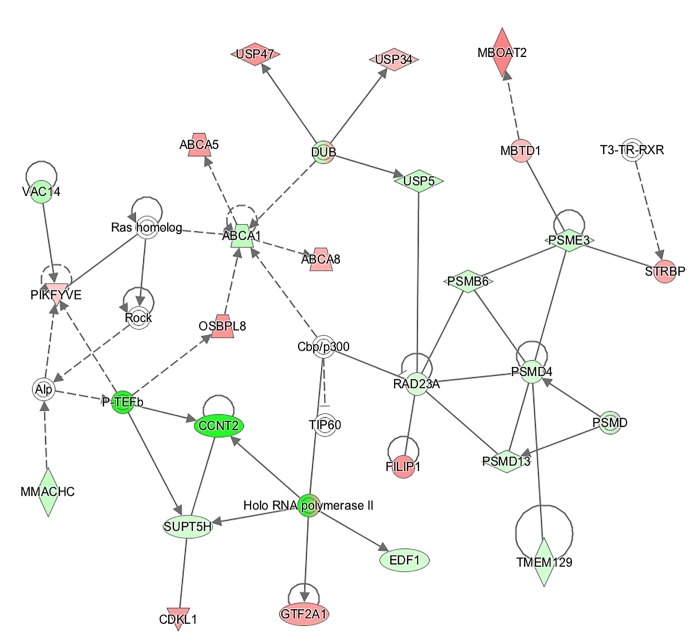
Connection of genes affecting functions related to “carbohydrate metabolism,” “lipid metabolism,” and “small molecule biochemistry” represented in a gene network (network 12). Biological relationship between genes is depicted as an edge/line (solid lines and dashed lines show direct and indirect interactions, respectively). Colors represent up- (red) and down- (green) regulated genes in high-feed efficient pigs.

89.2% of the revealed sequences were successfully mapped to the reference resulting in an average of 105.6 million high quality paired-end reads per sample assigned to 14,910 genes expressed in liver. A total of 922 genes were differentially expressed with a *P* < 0.01 (Figure 1) corresponding to false discovery rate (*q*) ≤ 0.16, and of these 818 were annotated (Figure 2 and Supplementary Table S1). Twenty-one molecular and cellular functions and twenty-one physiological system development and function categories were significantly enriched (*P* < 0.01) amongst the DE genes in relation to FE, as inferred from functional enrichment analysis (Supplementary Tables S2, S3). The highest distribution of all DE genes entries were observed in “cell death and survival” (12%), “cellular development” (11%), “organismal development” (11%), and “organismal survival” (10%). Twenty canonical pathways were significantly associated with DE genes in relation to FE at *P* < 0.01 (Table 4 and Supplementary Table S4), wherein the highest distribution of all DE gene entries were observed in “protein ubiquitination pathway” (8%) and “role of NFAT in regulation of the immune response” (8%), followed by “EIF2 signaling” (7%), “ILK signaling” (7%), “B cell receptor signaling” (7%), “aldosterone signaling in epithelial cells” (7%), and “gap junction signaling” (7%). Twenty-five networks were obtained upon integration of all DE genes. The most significant network (network 1) contained functions related to gastrointestinal and hepatic system disease and liver cirrhosis. Carbohydrate and lipid metabolism, and small molecule biochemistry were represented by 25 DE molecules in network 12 (Figure 3).

**Table 4:** Most significantly enriched canonical signaling pathways identified in liver samples of feed efficiency (FE)-divergent pigs.

Canonical pathways	-log(*p*-value)	Z-score	Gene
Role of NFAT in regulation of immune response	4.07	2.14^£^	***AKAP5***, ***AKT3***,***BLNK***, ***FRS2***, ***GAB1****, GNA11, GNAZ*, ***GNG2****, GSK3A*, ***ITPR1****, ITPR3*, ***MEF2A***, ***MEF2C***, ***MS4A2****, NFKBIB, ORAI1*,***PLCB1***, ***SOS1***, ***SOS2***
HGF signaling	3.36	2.33^£^	***AKT3****, CCND1*, ***ELF1***, ***ELF2****, ELF3*, ***ETS1***, ***FRS2***,***GAB1***, ***MAP3K1***, ***MAPK9***, ***MET***, ***SOS1***, ***SOS2***
Aldosterone signaling in epithelial cells	3.21	2.12^£^	***DNAJB4****, DNAJB12*, ***DNAJC6****, DNAJC11*, ***DNAJC13****, DNAJC16, DNAJC17, DNAJC22*, ***FRS2***, ***GAB1***, ***ITPR1****, ITPR3*, ***PIKFYVE***, ***PLCB1***, ***SOS1***,***SOS2***
Gap junction signaling	3.10	NA	***AKT3***, ***CAV1***, ***FRS2***, ***GAB1***, ***GUCY1B3***,***ITPR1****, ITPR3, NPR1*, ***PLCB1***, ***PRKG1***, ***SOS1***, ***SOS2****, TUBA1B, TUBB4B, TUBB, TUBG1*
Cell cycle regulation by BTG family proteins	2.73	NA	*CCND1, E2F1, E2F4, PPM1J, PRMT1*, ***PPP2R5A***
B cell receptor signaling	2.71	2.00	***AKT3***, ***BLNK****, CARD10*, ***ETS1***, ***FRS2***,***GAB1****, GSK3A*, ***MAP2K6***, ***MEF2C***, ***MAP3K1***, ***MAPK9****, NFKBIB*, ***PAG1***, ***PTEN***, ***SOS1***, ***SOS2***
tRNA charging	2.48	NA	*AARS, EARS2, FARSA, HARS, MARS, YARS*
ILK signaling	2.46	0.00	*ACTN1*, ***AKT3, BMP2****, CCND1*, ***FRS2***, ***GAB1****, GSK3A, KRT18*, ***MAPK9***, ***MAP2K6****, MYH9, PPP1R14B*, ***PPP2R5A***, ***PTEN****, PPM1J*, ***RICTOR***
14-3-3-mediated signaling	2.39	2.12^£^	***AKT3***, ***FRS2***, ***GAB1****, GSK3A*, ***MAPK9***, ***PLCB1****, STK11, TUBA1B, TUBB4B, TUBG1, TUBB, YWHAH*
EGF signaling	2.38	2.12^£^	***AKT3***, ***FRS2***, ***GAB1***, ***ITPR1****, ITPR3*, ***MAP3K1***, ***SOS1***, ***SOS2***

^£^*Significantly activated (z-score > 2) pathways in high-FE pigs; up-regulated genes in high-FE pigs are highlighted in bold and down-regulated genes in normal typeface; NA, no activity pattern available*.

### Verification of RNA-Seq Results

**Figure 4:**
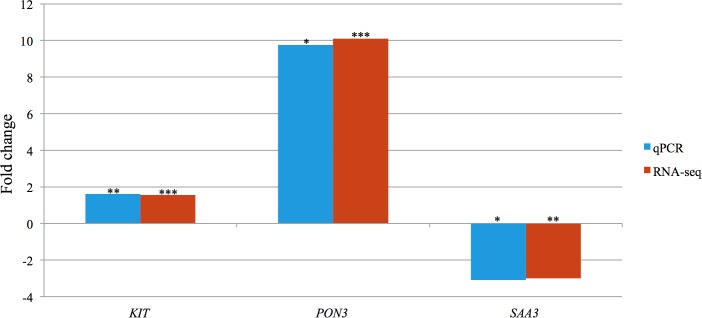
Bar chart portraying the RNA-seq and qPCR fold changes of three selected differentially expressed genes in high-FE pigs. Significance levels of differences affected by feed efficiency: ^∗^*P* < 0.05, ^∗∗^*P* < 0.01, ^∗∗∗^*P* < 0.001.

*KIT* (KIT proto-oncogene receptor tyrosine kinase), and *SAA3* (serum amyloid A3), selected randomly, as well as *PON3* (paraoxonase 3), selected based on its abundance, were amplified through qPCR. Significant differences in the expression levels of all three measured transcripts between the FE groups were confirmed. Spearman correlations attained by comparing gene expression levels measured using RNA-seq and qPCR, were found to be significant for *KIT* (*r* = 0.604, *P* < 0.05), *PON3* (*r* = 0.968, *P* < 0.001), and *SAA3* (*r* = 0.946, *P* < 0.001) (Figure 4).

## Discussion

In this study we investigated the hepatic transcriptome of pigs divergent in FE and identified a number of biological functions and pathways affiliated with lipid, protein and carbohydrate metabolism as well as hepatic growth and immune response. These differences help explain the physiological differences associated with divergence in FE and the recorded biochemical and hematological parameters.

### Macronutrients Metabolism

Paraoxonase 3 (*PON3*) was the most up-regulated (fold change = 10.1) gene in high-FE pigs. *PON3* codes for an enzyme that associates with HDL (Getz and Reardon, 2004) and prevents oxidation of LDL (Reddy et al., 2001) which otherwise would result in endothelial dysfunction (Leiva et al., 2015). Increased adipose deposition observed in PON3 knockout mice (Shih et al., 2015) is consistent with a potential role of PON3 in promoting lean growth and this is in keeping with decreased intramuscular fat content in the high-FE pigs found in our previous report (Horodyska et al., 2018a). Enrichment of DE genes in lipid metabolism networks was further portrayed by molecule connectivity analysis (Figure 3). Suppression of ATP binding cassette subfamily A member 1 (*ABCA1*) in mouse liver increased absorption of dietary cholesterol (Oram and Lawn, 2001). In the present study, the network illustrates *ABCA1* being down-regulated (fold change = -1.37) in high-FE pigs. Other members of the *ABCA* family (*ABCA5* and *ABCA8*) were over-expressed (fold change = 1.67 and 1.52, respectively) in high-FE pigs. In mice, abundance of *ABCA5* expression was previously associated with increased macrophage cholesterol efflux to HDL (Ye et al., 2010), whilst hepatic abundance of *ABCA8* led to significantly increased plasma HDL level and reverse cholesterol transport (Trigueros-Motos et al., 2017). Indeed, serum analysis pointed toward enhanced cholesterol level in high-FE pigs (Table 1). In addition, a positive correlation between serum cholesterol and total protein, which mainly constitutes of albumin, was observed. Interestingly, serum albumin mediates cholesterol efflux and may be a significant player in reverse cholesterol transport (Ha et al., 2003). Moreover, a previous study has also shown reverse cholesterol transport to be over-expressed in the livers of high-FE pigs (Gondret et al., 2017).

“Fibroblast growth factor (FGF) signaling,” which is involved in bile acid metabolism (Ornitz and Itoh, 2015), was predicted to be activated in high-FE livers (z-score = 3.00). In this pathway, fibroblast growth factor receptor substrate 2 (FRS2) was up-regulated (fold change = 1.31) in high-FE pigs. FRS2α deficiency led to increased bile acid synthesis in mouse liver (Wang et al., 2014) therefore it seems probable that high-FE pigs experience decreased bile acid production. In support of this, a previous study showed a lower abundance of genes involved in bile acid metabolism, nuclear receptor subfamily 1 group H member 4 (*NR1H4*) and squalene epoxidase (*SQLE*), in the liver of more feed efficient pigs (Reyer et al., 2017). Besides its well established functions, bile acids are also involved in lowering glucose levels (Staels and Fonseca, 2009) and hindering gluconeogenesis (Chai et al., 2015). Differential expression of cholesterol-related genes in the livers of pigs divergent for FE points toward increased absorption of dietary cholesterol and reverse cholesterol transport in high-FE pigs. Therefore, the inferred reduction in bile acid synthesis may be a measure to prevent drops in serum glucose level rather than explained by HDL cholesterol shortage. This presumption is in accordance with the higher glucose and cholesterol concentrations found in the serum of high-FE pigs. Gene ontology analysis also revealed “uptake and conversion of carbohydrates,” enclosed within a broader “carbohydrate metabolism” category, significantly enriched with DE genes and “aldosterone signaling in epithelial cells” significantly activated (z-score = 2.12) in high-FE pigs. Alongside its role in the regulation of sodium absorption (Briet and Schiffrin, 2010), aldosterone was shown to be involved in glucose transport through molecular regulation of SGLT1 (sodium-dependent glucose co-transporter) and GLUT2 (glucose transporter) in the chicken intestine (Garriga et al., 2001). The increased aldosterone signaling may indicate superior metabolic capacity in the liver of high-FE pigs compared to their counterparts (Grubbs et al., 2013).

The increased serum total protein concentration observed in high-FE pigs supports the suggested activation of “protein degradation and trafficking” sub-categories such as “protein catabolism and secretion” (z-score = 2.16 and 1.00, respectively) in the high-FE livers inferred from the functional enrichment analysis. Moreover, the greater serum protein concentration could have stimulated endogenous glucose synthesis (Promintzer and Krebs, 2006), which is consistent with the positive correlation between total protein and glucose concentrations in the serum of FE-divergent pigs. Over-expression of genes involved in protein synthesis and degradation have been reported in livers of high-FE pigs (Gondret et al., 2017). Lobley (2003) postulated that protein synthesis is much more energetically expensive in comparison to protein degradation. In our previous report (Horodyska et al., 2018b), we have suggested that high-FE muscle exhibits increased protein turnover and potentially reuses existing proteins, while directing the conserved energy toward more efficient growth. This phenomenon could also be occurring in the liver of high-FE pigs.

### Hepatic Growth

“Hepatocyte growth factor (HGF) signaling,” “epidermal growth factor (EGF) signaling,” and “FGF signaling” were significantly activated pathways (z-score = 2.33, 2.12, and 3.00, respectively) in high-FE pigs. Previous studies revealed a role for growth factors, e.g., HGF, EGF and FGF, in stimulating proliferation and differentiation of hepatic oval cells (Hu et al., 1993; Jones et al., 2009; Sánchez and Fabregat, 2010) and also in liver regeneration (Jiang et al., 1993; Steiling et al., 2003; Zimmers et al., 2017). Over-expression of growth factor receptor bound protein 2-associated protein 1 (*GAB1*) was a common feature shared between the three pathways. A study in GAB1 knockout mice reported defects in liver regeneration (Bard-Chapeau et al., 2006) and also reduced embryonic liver size (Sachs et al., 2000). AKT serine/threonine kinase 3 (*AKT3*) was another over-expressed gene enriched in these pathways. Akt3 is a member of AKT kinase family playing a role in modulation of cell survival and proliferation (Xu et al., 2012). Accordingly, “differentiation of epithelial cells,” enclosed within a “cellular development” function, was activated (z-score = 2.13). Moreover a “cell cycle” sub-category, “senescence of cells,” which is characterized by cell cycle arrest leading to loss of its ability to divide (Hoare et al., 2010), was suppressed (z-score = -2.90) in high-FE pigs. In the present study liver weights did not significantly differ between the FE groups, although a previous report found significantly heavier liver weights in high-FE pigs (Reyer et al., 2017). Cyclin T2 (*CCNT2*), coding for a protein regulating cell differentiation through activation of cyclin-dependent kinase 9 (CDK9) (Simone et al., 2002; Garriga et al., 2003), was the most down-regulated (fold change = -5.40) gene in high-FE pigs. CDK9 also functions in the inflammatory response (Han et al., 2014). Here, *CDK9* was down-regulated (fold change = -1.22) in high-FE pigs at a *P* < 0.05. It is possible that suppression of *CCNT2* could influence CDK9 function in differentiation of monocytes (De Falco et al., 2005) rather than hepatic epithelial cells.

### Immune Response

The “role of nuclear factor of activated T cells (NFAT) in regulation of the immune response” was significantly activated (z-score = 2.14) in high-FE pigs. NFAT proteins play a role in the first line of defense through regulating innate leukocyte response to inflammatory stimuli (Zanoni and Granucci, 2012). Myocyte enhancer factor 2C (*MEF2C*), which orchestrates immune cell activation and differentiation (Schuler et al., 2008), was enriched in this pathway and up-regulated in high-FE animals. Additionally, MEF2C belongs to a family of transcriptional factors that acts in conjunction with NFAT (Mancini and Toker, 2009). “Quantity of invariant natural killer T-cells” and “cell viability of natural killer cells,” falling under the broader “hematological system development and function” theme, were also significantly activated (z-score = 2.10 and 2.20, respectively) in high-FE pigs. In these sub-categories, ETS proto-oncogene 1 transcription factor (*ETS1*) and KIT proto-oncogene receptor tyrosine kinase (*KIT*) were up-regulated. ETS1 plays an essential role in the development and function of natural killer T cells, a group of cells exhibiting properties of both natural killer cells and T cells (Choi et al., 2011), whilst KIT is crucial for survival and maturation of natural killer cells (Colucci and Di Santo, 2000). Mutations within *KIT* gene are known to be pleiotropic, meaning that they affect several traits simultaneously and could cause expression bias in various tissues (Venhoranta et al., 2013; Gratten and Visscher, 2016). Therefore, although validation of *KIT* confirmed significant expression differences in FE-divergent liver, this gene needs to be considered with caution (Papakostas et al., 2014).

Consistent with the gene ontology, hematological analysis found an increased percentage of serum lymphocytes in the high-FE group. It is widely considered that during immune response dietary nutrients are shifted away from growth, which may lower animal’s FE, toward the immune-related processes (Patience et al., 2015). Nevertheless, a prompt response to hepatic pro-inflammatory stimuli may result in less energy consumed for combating systemic inflammation and hence more efficient utilization of nutrients for growth and protein accretion (Paradis et al., 2015). Several studies have reported a diverse hepatic inflammatory response in high- versus low-FE pigs (Gondret et al., 2017) and cattle (Alexandre et al., 2015; Paradis et al., 2015), thereby supporting this connection.

## Conclusion

Hepatic nutrient partitioning has a direct influence on the efficiency of energy utilization and potentially plays an important role in FE. In this study, carbohydrate, lipid and protein metabolism were significantly over-represented within the DE genes, confirming the hepatic influence on divergent energy utilization in high- versus low-FE pigs. In particular, high-FE pigs exhibited gene expression patterns suggesting improved hepatic absorption of carbohydrates and cholesterol as well as enhanced reverse cholesterol transport. Furthermore, the inferred decrease in bile acid synthesis in high-FE pigs may contribute to the increased concentrations of serum glucose observed. This increased glucose can be delivered to cells and utilized for increased growth. Gene ontology analysis also suggests that the liver of more feed efficient pigs may be characterized by higher protein turnover and increased epithelial cell differentiation, whilst enhanced quantity of invariant natural killer T-cells and viability of natural killer cells could induce a faster and more effective hepatic response to inflammatory stimuli.

## Availability of Data

RNA-seq data generated during the current study are available on ArrayExpress at EMBL-EBI (www.ebi.ac.uk/arrayexpress; accession number: E-MTAB-6256).

## Author Contributions

JH collected samples, extracted RNA, prepared libraries, validated RNA-seq via qPCR, carried out data analysis, and wrote the manuscript. RH conceived the experiment and contributed to experimental design, collected samples, and edited the manuscript. HR participated in statistical analysis and edited the manuscript. NT assisted in library preparation, performed the RNA-seq and data analysis, and edited the manuscript. PL provided the animals screened on RFI, participated in data collection and analysis, and edited the manuscript. UM determined serum and blood parameters, and edited the manuscript. KW contributed to experimental design, established lab protocols, and edited the manuscript.

## Conflict of Interest Statement

The authors declare that the research was conducted in the absence of any commercial or financial relationships that could be construed as a potential conflict of interest.

## Footnotes

1 www.R-project.org

2 www.ingenuity.com

3 https://www.ncbi.nlm.nih.gov/tools/primer-blast

4 http://www.ncbi.nlm.nih.gov/BLAST

## Abbreviations

FE: feed efficiency
HDL: high density lipoprotein
HRFI: high RFI
LDL: low density lipoprotein
LRFI: low RFI
RFI: residual feed intake.
