# Supplementary material for: RNA-Seq of Liver From Pigs Divergent in Feed Efficiency Highlights Shifts in Macronutrient Metabolism, Hepatic Growth and Immune Response
Source: Front Genet. 2019 Feb 19;10:117. doi: 10.3389/fgene.2019.00117 (PMC6389832; doi:10.3389/fgene.2019.00117)
Supplement: Supplementary file 1 [file Data_Sheet_1.PDF]

Supplementary material

RNA-seq of liver from pigs divergent in feed efficiency highlights shifts in macronutrient metabolism, hepatic growth and immune response

Justyna Horodyska, Ruth M. Hamill*, Henry Reyer, Nares Trakooljul, Peadar G. Lawlor, Ursula M. McCormack and Klaus Wimmers

**Corresponding author*

Table S1 Differentially expressed transcripts (n=818) at a P < 0.01 between high-FE and low-FE groups.

Gene Symbol	Gene Name	Fold Change	P-value	q-value
PON3	paraoxonase 3	10.1	2.99E-04	0.07
CPA1	carboxypeptidase A1	4.60	4.92E-04	0.08
APOB	apolipoprotein B	3.19	6.08E-03	0.15
KIAA0408	KIAA0408	2.96	1.04E-04	0.06
SFXN5	sideroflexin 5	2.80	4.42E-03	0.14
TNFAIP8L3	TNF alpha induced protein 8 like 3	2.75	1.66E-03	0.12
CATSPERB	cation channel sperm associated auxiliary subunit beta	2.58	2.63E-04	0.07
NAV3	neuron navigator 3	2.55	7.43E-04	0.10
CXCL11	C-X-C motif chemokine ligand 11	2.49	8.40E-03	0.16
BCAS1	breast carcinoma amplified sequence 1	2.46	2.80E-06	0.01
CDH6	cadherin 6	2.46	8.12E-05	0.06
SMC5	structural maintenance of chromosomes 5	2.44	9.14E-03	0.16
SGO2	shugoshin 2	2.43	4.89E-03	0.14
HORMAD1	HORMA domain containing 1	2.43	9.96E-03	0.16
FAM161A	family with sequence similarity 161 member A	2.40	6.21E-04	0.09
DNAJB4	DnaJ heat shock protein family (Hsp40) member B4	2.37	3.71E-03	0.14
NOX4	NADPH oxidase 4	2.35	5.18E-03	0.15
NES	nestin	2.34	4.17E-03	0.14
GPR174	G protein-coupled receptor 174	2.32	8.87E-03	0.16
PI15	peptidase inhibitor 15	2.31	5.36E-03	0.15
CLK1	CDC like kinase 1	2.23	6.28E-04	0.09
CEP290	centrosomal protein 290	2.22	5.45E-03	0.15
RPL15	ribosomal protein L15	2.21	8.57E-03	0.16
FAM171B	family with sequence similarity 171 member B	2.19	3.22E-04	0.07
CCDC91	coiled-coil domain containing 91	2.19	9.04E-03	0.16
GALR1	galanin receptor 1	2.18	6.40E-06	0.02
PCYOX1	prenylcysteine oxidase 1	2.18	2.79E-04	0.07
ICOS	inducible T-cell costimulator	2.17	7.08E-03	0.16
CD24	CD24 molecule	2.16	5.25E-04	0.08
CCDC82	coiled-coil domain containing 82	2.14	3.87E-03	0.14
CLK4	CDC like kinase 4	2.12	3.25E-04	0.07
CEP95	centrosomal protein 95	2.12	6.51E-03	0.15
GIN1	gypsy retrotransposon integrase 1	2.10	2.21E-03	0.13
CCDC18	coiled-coil domain containing 18	2.09	4.21E-03	0.14
CETN3	centrin 3	2.09	3.63E-03	0.14
ANGPTL3	angiopoietin like 3	2.08	3.51E-03	0.14
BIRC3	baculoviral IAP repeat containing 3	2.07	2.35E-03	0.13
PMAIP1	phorbol-12-myristate-13-acetate-induced protein 1	2.07	6.30E-04	0.09
N4BP2	NEDD4 binding protein 2	2.04	2.69E-03	0.14

AKAP5	A-kinase anchoring protein 5	2.04	6.30E-03	0.15
MYEF2	myelin expression factor 2	2.04	8.49E-03	0.16
LPAR6	lysophosphatidic acid receptor 6	2.02	7.53E-03	0.16
TRAT1	T-cell receptor associated transmembrane adaptor 1	2.01	2.33E-03	0.13
STK39	serine/threonine kinase 39	2.01	2.29E-03	0.13
SAMD9	sterile alpha motif domain containing 9	2.01	2.78E-04	0.07
PCM1	pericentriolar material 1	2.01	7.97E-03	0.16
MUM1L1	MUM1 like 1	2.00	3.18E-03	0.14
ZNF638	zinc finger protein 638	1.99	8.83E-03	0.16
AKAP9	A-kinase anchoring protein 9	1.99	3.64E-03	0.14
SENP6	SUMO1/sentrin specific peptidase 6	1.99	7.18E-03	0.16
TRAPPC8	trafficking protein particle complex 8	1.98	5.91E-03	0.15
TET1	tet methylcytosine dioxygenase 1	1.97	2.07E-04	0.07
MSANTD4	Myb/SANT DNA binding domain containing 4 with coiled-coils	1.97	5.90E-03	0.15
SPARCL1	SPARC like 1	1.97	1.49E-04	0.07
ANGPT2	angiopoietin 2	1.95	6.01E-05	0.05
SEPSECS	Sep (O-phosphoserine) tRNA:Sec (selenocysteine) tRNA synthase	1.95	4.51E-03	0.14
TMEM71	transmembrane protein 71	1.94	9.78E-03	0.16
ARHGAP15	Rho GTPase activating protein 15	1.93	2.45E-03	0.13
GULP1	GULP, engulfment adaptor PTB domain containing 1	1.93	6.54E-03	0.15
TAX1BP1	Tax1 binding protein 1	1.93	6.17E-03	0.15
C1QTNF7	C1q and TNF related 7	1.93	7.67E-03	0.16
ZFP30	ZFP30 zinc finger protein	1.92	3.60E-03	0.14
KLHL23/PHOSPHO2-KLHL23	kelch like family member 23	1.92	7.59E-03	0.16
PDS5B	PDS5 cohesin associated factor B	1.92	4.05E-03	0.14
PRTFDC1	phosphoribosyl transferase domain containing 1	1.92	6.47E-03	0.15
LUC7L3	LUC7 like 3 pre-mRNA splicing factor	1.91	9.44E-03	0.16
NAE1	NEDD8 activating enzyme E1 subunit 1	1.91	7.43E-03	0.16
ACTR6	ARP6 actin-related protein 6 homolog	1.91	7.86E-03	0.16
HFM1	HFM1, ATP dependent DNA helicase homolog	1.91	1.60E-03	0.12
GPR52	G protein-coupled receptor 52	1.90	4.85E-03	0.14
JMJD1C	jumonji domain containing 1C	1.90	1.34E-03	0.12
WSB1	WD repeat and SOCS box containing 1	1.90	1.40E-03	0.12
CD36	CD36 molecule	1.90	4.88E-03	0.14
CH25H	cholesterol 25-hydroxylase	1.89	5.53E-03	0.15
U2SURP	U2 snRNP associated SURP domain containing	1.89	3.20E-03	0.14
RBM46	RNA binding motif protein 46	1.89	8.23E-03	0.16
ZC3H6	zinc finger CCCH-type containing 6	1.89	1.62E-03	0.12
CCDC186	coiled-coil domain containing 186	1.89	7.51E-03	0.16
ZNF382	zinc finger protein 382	1.89	7.14E-03	0.16
NT5C3A	5'-nucleotidase, cytosolic IIIA	1.89	5.07E-03	0.15
ZNF770	zinc finger protein 770	1.89	9.29E-03	0.16
CBX3	chromobox 3	1.89	9.58E-03	0.16
SLCO4C1	solute carrier organic anion transporter family member 4C1	1.88	6.69E-03	0.15
PRPF39	pre-mRNA processing factor 39	1.88	3.20E-03	0.14
PPIP5K2	diphosphoinositol pentakisphosphate kinase 2	1.88	5.22E-03	0.15
OGN	osteoglycin	1.87	1.82E-03	0.13
MBOAT2	membrane bound O-acyltransferase domain containing 2	1.87	7.10E-03	0.16
THNSL1	threonine synthase like 1	1.86	4.88E-03	0.14
FXR1	FMR1 autosomal homolog 1	1.86	6.90E-03	0.16
KLHL7	kelch like family member 7	1.86	4.57E-03	0.14
ZFC3H1	zinc finger C3H1-type containing	1.86	4.56E-03	0.14

CUL4B	cullin 4B	1.85	6.57E-03	0.15
MYO5C	myosin VC	1.85	2.21E-04	0.07
ARHGAP5	Rho GTPase activating protein 5	1.85	4.85E-03	0.14
EPHA7	EPH receptor A7	1.85	1.17E-03	0.11
STAT4	signal transducer and activator of transcription 4	1.85	6.29E-03	0.15
RAVER2	ribonucleoprotein, PTB binding 2	1.85	5.98E-03	0.15
ITPR1	inositol 1,4,5-trisphosphate receptor type 1	1.85	4.05E-03	0.14
PNPLA8	patatin like phospholipase domain containing 8	1.85	5.19E-03	0.15
PJA2	praja ring finger ubiquitin ligase 2	1.85	3.84E-03	0.14
EMCN	endomucin	1.84	3.59E-03	0.14
DEK	DEK proto-oncogene	1.84	3.56E-03	0.14
NKTR	natural killer cell triggering receptor	1.84	7.64E-03	0.16
ZFP14	ZFP14 zinc finger protein	1.84	8.34E-03	0.16
RSF1	remodeling and spacing factor 1	1.84	3.93E-03	0.14
RBM48	RNA binding motif protein 48	1.83	3.05E-03	0.14
KITLG	KIT ligand	1.83	4.99E-03	0.15
PDE1A	phosphodiesterase 1A	1.83	1.95E-04	0.07
FBXL17	F-box and leucine rich repeat protein 17	1.83	3.08E-03	0.14
SCTR	secretin receptor	1.82	2.83E-03	0.14
FNIP1	folliculin interacting protein 1	1.82	9.35E-04	0.10
CD69	CD69 molecule	1.81	1.84E-03	0.13
BHLHB9	basic helix-loop-helix family member b9	1.80	6.35E-05	0.05
MAMDC2	MAM domain containing 2	1.80	8.00E-03	0.16
PI16	peptidase inhibitor 16	1.80	1.34E-03	0.12
URI1	URI1, prefoldin like chaperone	1.79	9.74E-03	0.16
CEP152	centrosomal protein 152	1.79	7.78E-03	0.16
HESX1	HESX homeobox 1	1.78	8.81E-03	0.16
PNRC1	proline rich nuclear receptor coactivator 1	1.78	1.75E-03	0.12
MYSM1	Myb like, SWIRM and MPN domains 1	1.78	5.53E-03	0.15
SKIL	SKI like proto-oncogene	1.78	6.03E-03	0.15
FLRT3	fibronectin leucine rich transmembrane protein 3	1.77	2.97E-04	0.07
ZNF793	zinc finger protein 793	1.77	1.76E-05	0.03
TMOD2	tropomodulin 2	1.77	3.49E-03	0.14
ZNF189	zinc finger protein 189	1.76	8.67E-03	0.16
BAZ2B	bromodomain adjacent to zinc finger domain 2B	1.76	3.71E-03	0.14
THAP2	THAP domain containing 2	1.76	8.46E-03	0.16
ZNF555	zinc finger protein 555	1.76	9.85E-03	0.16
DNAJC6	DnaJ heat shock protein family (Hsp40) member C6	1.76	4.78E-03	0.14
SLU7	SLU7 homolog, splicing factor	1.75	8.57E-03	0.16
NR4A2	nuclear receptor subfamily 4 group A member 2	1.75	5.72E-03	0.15
NUGGC	nuclear GTPase, germinal center associated	1.75	2.31E-04	0.07
GAS2	growth arrest specific 2	1.75	2.37E-03	0.13
FGD4	FYVE, RhoGEF and PH domain containing 4	1.74	8.04E-03	0.16
RSBN1	round spermatid basic protein 1	1.74	4.05E-03	0.14
PTBP2	polypyrimidine tract binding protein 2	1.74	2.44E-03	0.13
RBM12B	RNA binding motif protein 12B	1.74	8.46E-03	0.16
ASPN	asporin	1.74	3.79E-03	0.14
SMCHD1	structural maintenance of chromosomes flexible hinge domain containing 1	1.74	6.41E-03	0.15
ICE2	interactor of little elongation complex ELL subunit 2	1.74	5.83E-03	0.15
CEP63	centrosomal protein 63	1.74	5.52E-03	0.15
FNBP4	formin binding protein 4	1.74	4.49E-03	0.14
SMC3	structural maintenance of chromosomes 3	1.74	7.88E-03	0.16
GBP5	guanylate binding protein 5	1.74	1.23E-03	0.11
ELF2	E74 like ETS transcription factor 2	1.74	3.61E-03	0.14
GPR183	G protein-coupled receptor 183	1.74	8.06E-03	0.16

RALGAPA1	Ral GTPase activating protein catalytic alpha subunit 1	1.74	8.57E-04	0.10
CSPP1	centrosome and spindle pole associated protein 1	1.74	1.40E-03	0.12
ZNF827	zinc finger protein 827	1.73	7.27E-03	0.16
USP47	ubiquitin specific peptidase 47	1.73	3.59E-03	0.14
APLF	aprataxin and PNKP like factor	1.73	4.88E-03	0.14
ACAP2	ArfGAP with coiled-coil, ankyrin repeat and PH domains 2	1.73	4.60E-03	0.14
FILIP1	filamin A interacting protein 1	1.73	4.81E-03	0.14
NFYB	nuclear transcription factor Y subunit beta	1.73	9.12E-03	0.16
EXPH5	exophilin 5	1.72	2.27E-03	0.13
CFTR	cystic fibrosis transmembrane conductance regulator	1.72	5.51E-05	0.05
GRAMD1C	GRAM domain containing 1C	1.72	9.76E-03	0.16
ETV1	ETS variant 1	1.71	4.31E-04	0.08
DPP4	dipeptidyl peptidase 4	1.71	3.24E-03	0.14
TOP2B	topoisomerase (DNA) II beta	1.71	9.68E-03	0.16
ZNF12	zinc finger protein 12	1.71	5.57E-03	0.15
PDK4	pyruvate dehydrogenase kinase 4	1.71	8.49E-03	0.16
SLCO1A2	solute carrier organic anion transporter family member 1A2	1.70	2.90E-03	0.14
DIAPH2	diaphanous related formin 2	1.70	3.35E-04	0.07
VEPH1	ventricular zone expressed PH domain containing 1	1.70	3.28E-04	0.07
OSBPL8	oxysterol binding protein like 8	1.70	9.23E-03	0.16
MXI1	MAX interactor 1, dimerization protein	1.70	3.55E-03	0.14
CREBRF	CREB3 regulatory factor	1.69	3.62E-03	0.14
MED13	mediator complex subunit 13	1.69	7.64E-03	0.16
DZIP3	DAZ interacting zinc finger protein 3	1.69	3.97E-03	0.14
GEM	GTP binding protein overexpressed in skeletal muscle	1.69	4.72E-03	0.14
TRIP11	thyroid hormone receptor interactor 11	1.69	9.71E-03	0.16
PYROXD1	pyridine nucleotide-disulphide oxidoreductase domain 1	1.69	8.00E-03	0.16
MITD1	microtubule interacting and trafficking domain containing 1	1.69	6.08E-03	0.15
ZNF81	zinc finger protein 81	1.69	5.64E-03	0.15
CCNG2	cyclin G2	1.68	1.07E-04	0.06
SCAPER	S-phase cyclin A associated protein in the ER	1.68	6.63E-03	0.15
PHF14	PHD finger protein 14	1.68	7.36E-03	0.16
STAG2	stromal antigen 2	1.68	4.20E-03	0.14
ARAP2	ArfGAP with RhoGAP domain, ankyrin repeat and PH domain 2	1.68	1.33E-03	0.12
ZNF473	zinc finger protein 473	1.68	3.37E-03	0.14
ECM2	extracellular matrix protein 2	1.68	1.89E-04	0.07
HNMT	histamine N-methyltransferase	1.68	3.70E-03	0.14
KLRC1	killer cell lectin like receptor C1	1.68	3.71E-03	0.14
ZNF277	zinc finger protein 277	1.68	4.06E-04	0.08
MECOM	MDS1 and EVI1 complex locus	1.68	2.92E-03	0.14
NCOA7	nuclear receptor coactivator 7	1.68	4.77E-04	0.08
TGFB2	transforming growth factor beta 2	1.67	9.03E-04	0.10
EPM2AIP1	EPM2A interacting protein 1	1.67	4.80E-03	0.14
ABCA5	ATP binding cassette subfamily A member 5	1.67	7.31E-04	0.10
KCNJ16	potassium voltage-gated channel subfamily J member 16	1.67	8.49E-03	0.16
FAM135A	family with sequence similarity 135 member A	1.67	8.78E-03	0.16
ARID4B	AT-rich interaction domain 4B	1.67	6.92E-03	0.16
RFTN2	raftlin family member 2	1.67	2.15E-03	0.13
TTC37	tetratricopeptide repeat domain 37	1.66	7.28E-03	0.16
CCDC88A	coiled-coil domain containing 88A	1.66	8.26E-03	0.16

BMP2	bone morphogenetic protein 2	1.66	3.81E-03	0.14
TTC14	tetratricopeptide repeat domain 14	1.66	3.95E-03	0.14
STAG1	stromal antigen 1	1.66	4.71E-03	0.14
KIAA2026	KIAA2026	1.66	4.86E-04	0.08
CDKL1	cyclin dependent kinase like 1	1.66	2.30E-03	0.13
DMP1	dentin matrix acidic phosphoprotein 1	1.65	8.84E-03	0.16
RYBP	RING1 and YY1 binding protein	1.65	3.06E-03	0.14
ERBIN	erbb2 interacting protein	1.65	7.44E-03	0.16
CAV1	caveolin 1	1.65	5.29E-03	0.15
STX2	syntaxin 2	1.65	4.35E-03	0.14
SCAF11	SR-related CTD associated factor 11	1.65	8.18E-03	0.16
MEF2A	myocyte enhancer factor 2A	1.65	3.49E-03	0.14
ITGA8	integrin subunit alpha 8	1.65	4.16E-04	0.08
IGFBP5	insulin like growth factor binding protein 5	1.65	7.49E-03	0.16
HHLA2	HERV-H LTR-associating 2	1.65	3.56E-03	0.14
BIRC2	baculoviral IAP repeat containing 2	1.64	9.54E-03	0.16
STRN3	striatin 3	1.64	7.61E-03	0.16
ATXN3L	ataxin 3 like	1.64	3.52E-03	0.14
FBXO32	F-box protein 32	1.64	1.52E-03	0.12
RAB11FIP2	RAB11 family interacting protein 2	1.64	2.36E-03	0.13
KCNMA1	potassium calcium-activated channel subfamily M alpha 1	1.63	2.41E-03	0.13
GTF2A1	general transcription factor IIA subunit 1	1.63	7.97E-03	0.16
ABCA9	ATP binding cassette subfamily A member 9	1.63	6.47E-03	0.15
RNF125	ring finger protein 125	1.63	8.75E-03	0.16
TBC1D15	TBC1 domain family member 15	1.62	9.28E-03	0.16
NIPSNAP3B	nipsnap homolog 3B	1.62	3.01E-04	0.07
ZNF280D	zinc finger protein 280D	1.62	7.00E-03	0.16
PRKG1	protein kinase, cGMP-dependent, type I	1.62	1.30E-03	0.12
NFE2L2	nuclear factor, erythroid 2 like 2	1.62	7.13E-04	0.10
FAP	fibroblast activation protein alpha	1.61	3.79E-04	0.08
EIF3E	eukaryotic translation initiation factor 3 subunit E	1.61	4.53E-03	0.14
ANKRD12	ankyrin repeat domain 12	1.61	5.05E-03	0.15
LAMA4	laminin subunit alpha 4	1.61	7.11E-05	0.05
UHRF1BP1L	UHRF1 binding protein 1 like	1.61	1.51E-03	0.12
MAP4K5	mitogen-activated protein kinase kinase kinase kinase 5	1.61	9.47E-04	0.10
LRIG2	leucine rich repeats and immunoglobulin like domains 2	1.61	1.25E-03	0.11
TRIM2	tripartite motif containing 2	1.61	3.93E-03	0.14
EVI5	ecotropic viral integration site 5	1.61	5.29E-03	0.15
PLOD2	procollagen-lysine,2-oxoglutarate 5-dioxygenase 2	1.60	8.79E-03	0.16
ZNF569	zinc finger protein 569	1.60	8.82E-03	0.16
SLC10A5	solute carrier family 10 member 5	1.60	1.60E-03	0.12
GOLGB1	golgin B1	1.60	4.30E-03	0.14
CD300C	CD300c molecule	1.60	1.13E-03	0.11
NOSTRIN	nitric oxide synthase trafficking	1.59	4.07E-03	0.14
N4BP2L2	NEDD4 binding protein 2 like 2	1.59	5.74E-03	0.15
RNF19A	ring finger protein 19A, RBR E3 ubiquitin protein ligase	1.59	4.11E-03	0.14
TC2N	tandem C2 domains, nuclear	1.59	1.02E-03	0.11
APC	APC, WNT signaling pathway regulator	1.59	5.42E-03	0.15
SECISBP2L	SECIS binding protein 2 like	1.59	9.23E-03	0.16
MAP2K6	mitogen-activated protein kinase kinase 6	1.59	8.65E-03	0.16
ATP8B1	ATPase phospholipid transporting 8B1	1.59	2.52E-03	0.14
RYR2	ryanodine receptor 2	1.58	3.03E-03	0.14
ARHGAP29	Rho GTPase activating protein 29	1.58	2.63E-04	0.07

CEP162	centrosomal protein 162	1.58	8.56E-03	0.16
STRBP	spermatid perinuclear RNA binding protein	1.58	3.14E-03	0.14
EML4	echinoderm microtubule associated protein like 4	1.58	7.99E-03	0.16
SMARCA1	SWI/SNF related, matrix associated, actin dependent regulator of chromatin, subfamily a, member 1	1.57	5.67E-03	0.15
ZNF84	zinc finger protein 84	1.57	2.21E-03	0.13
TSPAN12	tetraspanin 12	1.57	8.37E-04	0.10
DDX60	DExD/H-box helicase 60	1.57	7.08E-03	0.16
PRPF18	pre-mRNA processing factor 18	1.57	4.84E-03	0.14
METTL25	methyltransferase like 25	1.57	8.77E-03	0.16
RUFY2	RUN and FYVE domain containing 2	1.57	7.54E-03	0.16
CNOT10	CCR4-NOT transcription complex subunit 10	1.57	5.68E-03	0.15
KIAA1109	KIAA1109	1.56	8.89E-03	0.16
RFC1	replication factor C subunit 1	1.56	3.60E-03	0.14
ZCCHC11	zinc finger CCHC-type containing 11	1.56	7.44E-03	0.16
CDC14B	cell division cycle 14B	1.56	7.15E-04	0.10
MYO5A	myosin VA	1.56	6.54E-04	0.10
BBS12	Bardet-Biedl syndrome 12	1.56	9.16E-03	0.16
NME7	NME/NM23 family member 7	1.56	3.37E-03	0.14
KIT	KIT proto-oncogene receptor tyrosine kinase	1.56	1.12E-06	0.01
MS4A2	membrane spanning 4-domains A2	1.56	9.18E-03	0.16
ARID4A	AT-rich interaction domain 4A	1.55	4.27E-03	0.14
CALCRL	calcitonin receptor like receptor	1.55	7.15E-03	0.16
RWDD3	RWD domain containing 3	1.55	4.81E-03	0.14
ZNF280C	zinc finger protein 280C	1.55	6.53E-03	0.15
SP4	Sp4 transcription factor	1.55	4.61E-04	0.08
HCFC2	host cell factor C2	1.55	7.00E-03	0.16
ASH1L	ASH1 like histone lysine methyltransferase	1.55	6.55E-03	0.15
RLF	rearranged L-myc fusion	1.55	8.14E-03	0.16
RIDA	reactive intermediate imine deaminase A homolog	1.55	9.70E-03	0.16
GBP4	guanylate binding protein 4	1.55	1.66E-03	0.12
KLHL6	kelch like family member 6	1.54	5.61E-03	0.15
OSGEPL1	O-sialoglycoprotein endopeptidase like 1	1.54	7.64E-03	0.16
SAFB2	scaffold attachment factor B2	1.54	1.29E-03	0.12
SLPI	secretory leukocyte peptidase inhibitor	1.54	3.52E-03	0.14
MEF2C	myocyte enhancer factor 2C	1.54	4.14E-04	0.08
NSL1	NSL1, MIS12 kinetochore complex component	1.54	3.28E-03	0.14
SP100	SP100 nuclear antigen	1.53	8.69E-03	0.16
SOX4	SRY-box 4	1.53	9.65E-03	0.16
NAALADL2	N-acetylated alpha-linked acidic dipeptidase like 2	1.53	4.25E-03	0.14
NPHP1	nephrocystin 1	1.53	6.96E-03	0.16
FCHO2	FCH domain only 2	1.53	8.06E-03	0.16
SYNE1	spectrin repeat containing nuclear envelope protein 1	1.53	2.95E-03	0.14
ALG13	ALG13, UDP-N-acetylglucosaminyltransferase subunit	1.53	9.25E-03	0.16
DPY19L4	dpy-19 like 4 (C. elegans)	1.53	6.69E-03	0.15
FLRT2	fibronectin leucine rich transmembrane protein 2	1.53	2.26E-04	0.07
VRK2	vaccinia related kinase 2	1.53	5.67E-03	0.15
NBEAL1	neurobeachin like 1	1.53	3.52E-03	0.14
TMEM57	transmembrane protein 57	1.52	8.58E-03	0.16
MET	MET proto-oncogene, receptor tyrosine kinase	1.52	8.78E-03	0.16
CYLD	CYLD lysine 63 deubiquitinase	1.52	8.54E-03	0.16
ARSK	arylsulfatase family member K	1.52	3.53E-03	0.14
ABCA8	ATP binding cassette subfamily A member 8	1.52	5.52E-03	0.15
PLD1	phospholipase D1	1.52	6.92E-04	0.10
RPRD1A	regulation of nuclear pre-mRNA domain containing 1A	1.51	7.43E-03	0.16

MSANTD2	Myb/SANT DNA binding domain containing 2	1.51	9.92E-03	0.16
GTF3C3	general transcription factor IIIC subunit 3	1.51	8.49E-03	0.16
ZNF606	zinc finger protein 606	1.51	2.98E-03	0.14
KLF3	Kruppel like factor 3	1.51	3.54E-03	0.14
MARCKS	myristoylated alanine rich protein kinase C substrate	1.50	5.44E-03	0.15
ABCC9	ATP binding cassette subfamily C member 9	1.50	2.17E-03	0.13
GUCY1B3	guanylate cyclase 1 soluble subunit beta	1.50	3.85E-03	0.14
SPG11	SPG11, spatacsin vesicle trafficking associated	1.50	6.42E-03	0.15
FBXO39	F-box protein 39	1.50	9.26E-03	0.16
SEMA3D	semaphorin 3D	1.50	5.26E-03	0.15
SHE	Src homology 2 domain containing E	1.50	6.93E-03	0.16
ZNF268	zinc finger protein 268	1.50	9.02E-03	0.16
ANKRD50	ankyrin repeat domain 50	1.50	1.05E-03	0.11
HNRNPH3	heterogeneous nuclear ribonucleoprotein H3	1.49	2.11E-03	0.13
DNAJC13	DnaJ heat shock protein family (Hsp40) member C13	1.49	9.54E-03	0.16
PDE8B	phosphodiesterase 8B	1.49	1.48E-03	0.12
ARHGEF3	Rho guanine nucleotide exchange factor 3	1.49	2.81E-03	0.14
GABPB1	GA binding protein transcription factor beta subunit 1	1.49	4.72E-03	0.14
CCNG1	cyclin G1	1.49	3.84E-03	0.14
ESCO1	establishment of sister chromatid cohesion N-acetyltransferase 1	1.49	1.31E-03	0.12
SDR9C7	short chain dehydrogenase/reductase family 9C member 7	1.49	1.19E-03	0.11
MGEA5	meningioma expressed antigen 5 (hyaluronidase)	1.49	6.07E-03	0.15
ZNF514	zinc finger protein 514	1.49	2.79E-03	0.14
ZNF292	zinc finger protein 292	1.48	1.54E-03	0.12
KIAA1429	KIAA1429	1.48	4.50E-03	0.14
KLF4	Kruppel like factor 4	1.48	6.43E-03	0.15
TRPM7	transient receptor potential cation channel subfamily M member 7	1.48	3.99E-03	0.14
LHFP	lipoma HMGIC fusion partner	1.48	6.27E-03	0.15
LGR4	leucine rich repeat containing G protein-coupled receptor 4	1.48	2.08E-04	0.07
STON1	stonin 1	1.48	9.51E-03	0.16
ALPK1	alpha kinase 1	1.47	1.45E-03	0.12
SOS2	SOS Ras/Rho guanine nucleotide exchange factor 2	1.47	6.33E-03	0.15
PLK2	polo like kinase 2	1.47	1.09E-03	0.11
RBM26	RNA binding motif protein 26	1.47	6.87E-03	0.16
ZDHHC6	zinc finger DHHC-type containing 6	1.47	6.98E-03	0.16
NIN	ninein	1.47	9.20E-03	0.16
UBR3	ubiquitin protein ligase E3 component n-recognin 3 (putative)	1.47	9.68E-03	0.16
HECA	hdc homolog, cell cycle regulator	1.47	5.66E-03	0.15
PODXL	podocalyxin like	1.46	3.88E-03	0.14
RICTOR	RPTOR independent companion of MTOR complex 2	1.46	1.54E-03	0.12
CAT	catalase	1.46	6.73E-03	0.15
RNASEL	ribonuclease L	1.46	3.63E-03	0.14
PAG1	phosphoprotein membrane anchor with glycosphingolipid microdomains 1	1.46	2.16E-03	0.13
SYNE2	spectrin repeat containing nuclear envelope protein 2	1.46	9.71E-03	0.16
CXorf23	chromosome X open reading frame 23	1.45	2.23E-03	0.13
PROCR	protein C receptor	1.45	6.80E-03	0.16
MAPK9	mitogen-activated protein kinase 9	1.45	8.69E-03	0.16
ABI3BP	ABI family member 3 binding protein	1.45	5.08E-03	0.15
GCNT1	glucosaminyl (N-acetyl) transferase 1, core 2	1.45	7.39E-03	0.16
RASGRP1	RAS guanyl releasing protein 1	1.44	1.66E-04	0.07
RFX7	regulatory factor X7	1.44	3.79E-03	0.14

ATG4C	autophagy related 4C cysteine peptidase	1.44	5.81E-03	0.15
PPP2R5A	protein phosphatase 2 regulatory subunit B'alpha	1.44	4.27E-03	0.14
CD84	CD84 molecule	1.44	5.81E-03	0.15
SPAG9	sperm associated antigen 9	1.44	3.00E-03	0.14
LGALS8	galectin 8	1.44	6.48E-03	0.15
FBXO3	F-box protein 3	1.44	9.05E-03	0.16
BLNK	B-cell linker	1.44	8.39E-03	0.16
LRRK2	leucine rich repeat kinase 2	1.43	7.00E-03	0.16
S1PR1	sphingosine-1-phosphate receptor 1	1.43	8.08E-03	0.16
LAMB4	laminin subunit beta 4	1.43	4.25E-03	0.14
MBTD1	mbt domain containing 1	1.43	2.70E-03	0.14
SOS1	SOS Ras/Rac guanine nucleotide exchange factor 1	1.43	5.20E-03	0.15
USP34	ubiquitin specific peptidase 34	1.43	5.98E-03	0.15
PEX1	peroxisomal biogenesis factor 1	1.43	6.31E-03	0.15
HPSE	heparanase	1.42	4.14E-03	0.14
TMTC1	transmembrane and tetratricopeptide repeat containing 1	1.42	8.81E-03	0.16
DHX40	DEAH-box helicase 40	1.42	8.77E-03	0.16
RBPJ	recombination signal binding protein for immunoglobulin kappa J region	1.42	9.34E-03	0.16
SNRK	SNF related kinase	1.42	1.73E-03	0.12
TMEM106B	transmembrane protein 106B	1.42	5.21E-03	0.15
FYB	FYN binding protein	1.42	4.29E-03	0.14
FRMD4B	FERM domain containing 4B	1.42	3.66E-03	0.14
FBXL3	F-box and leucine rich repeat protein 3	1.42	8.57E-03	0.16
SLIT2	slit guidance ligand 2	1.42	1.83E-03	0.13
FAR1	fatty acyl-CoA reductase 1	1.41	8.37E-03	0.16
PTEN	phosphatase and tensin homolog	1.41	8.10E-03	0.16
NCOA1	nuclear receptor coactivator 1	1.41	1.99E-03	0.13
HELZ	helicase with zinc finger	1.41	1.77E-03	0.13
MSL2	male-specific lethal 2 homolog (Drosophila)	1.41	4.38E-03	0.14
VPS13B	vacuolar protein sorting 13 homolog B	1.41	4.84E-03	0.14
VPS8	VPS8, CORVET complex subunit	1.41	5.05E-03	0.15
TIAM1	T-cell lymphoma invasion and metastasis 1	1.41	5.52E-03	0.15
GRB14	growth factor receptor bound protein 14	1.41	4.56E-03	0.14
RBL2	RB transcriptional corepressor like 2	1.40	3.95E-03	0.14
CD2	CD2 molecule	1.40	6.00E-03	0.15
PLSCR4	phospholipid scramblase 4	1.40	9.01E-03	0.16
TMEM19	transmembrane protein 19	1.40	7.62E-03	0.16
IL10RA	interleukin 10 receptor subunit alpha	1.40	3.99E-03	0.14
ITGA1	integrin subunit alpha 1	1.39	2.96E-03	0.14
KDM7A	lysine demethylase 7A	1.39	4.83E-03	0.14
CREG1	cellular repressor of E1A stimulated genes 1	1.39	8.62E-03	0.16
ANXA4	annexin A4	1.39	2.26E-04	0.07
BMPER	BMP binding endothelial regulator	1.38	2.62E-03	0.14
ERAP2	endoplasmic reticulum aminopeptidase 2	1.38	7.15E-03	0.16
TMEM87B	transmembrane protein 87B	1.37	8.00E-03	0.16
CBLB	Cbl proto-oncogene B	1.37	7.83E-05	0.06
ANKMY2	ankyrin repeat and MYND domain containing 2	1.37	8.53E-03	0.16
FAM117B	family with sequence similarity 117 member B	1.37	1.64E-03	0.12
HPS3	HPS3, biogenesis of lysosomal organelles complex 2 subunit 1	1.37	2.09E-03	0.13
GREB1L	growth regulation by estrogen in breast cancer 1 like	1.36	2.09E-04	0.07
RAPGEF4	Rap guanine nucleotide exchange factor 4	1.36	4.25E-03	0.14
KANSL1L	KAT8 regulatory NSL complex subunit 1 like	1.36	2.20E-03	0.13
SUCLG2	succinate-CoA ligase GDP-forming beta subunit	1.36	8.94E-03	0.16
INTS6L	integrator complex subunit 6 like	1.36	4.73E-03	0.14

CMAHP	cytidine monophospho-N-acetylneuraminic acid hydroxylase, pseudogene	1.36	5.20E-03	0.15
RIPK2	receptor interacting serine/threonine kinase 2	1.35	9.01E-03	0.16
GNG2	G protein subunit gamma 2	1.35	8.15E-03	0.16
CPEB4	cytoplasmic polyadenylation element binding protein 4	1.35	2.71E-05	0.04
ZBTB37	zinc finger and BTB domain containing 37	1.34	1.83E-03	0.13
UTRN	utrophin	1.34	5.23E-03	0.15
ADGRL2	adhesion G protein-coupled receptor L2	1.34	7.74E-03	0.16
ERCC6L2	ERCC excision repair 6 like 2	1.34	6.82E-03	0.16
AKT3	AKT serine/threonine kinase 3	1.33	6.77E-03	0.16
TAB2	TGF-beta activated kinase 1/MAP3K7 binding protein 2	1.33	7.82E-03	0.16
ARMCX5-GPRASP2/GPRASP2	G protein-coupled receptor associated sorting protein 2	1.33	1.61E-03	0.12
NCALD	neurocalcin delta	1.33	4.56E-03	0.14
DDHD2	DDHD domain containing 2	1.33	4.71E-03	0.14
GIMAP8	GTPase, IMAP family member 8	1.33	4.87E-03	0.14
MAP3K1	mitogen-activated protein kinase kinase kinase 1	1.33	3.32E-03	0.14
GAB1	GRB2 associated binding protein 1	1.32	1.41E-03	0.12
TBC1D32	TBC1 domain family member 32	1.32	3.99E-04	0.08
RMND5A	required for meiotic nuclear division 5 homolog A	1.32	9.83E-03	0.16
RNF38	ring finger protein 38	1.32	3.38E-04	0.07
FAM126A	family with sequence similarity 126 member A	1.32	4.33E-03	0.14
ZEB2	zinc finger E-box binding homeobox 2	1.31	2.39E-03	0.13
CKAP5	cytoskeleton associated protein 5	1.31	8.61E-03	0.16
FRS2	fibroblast growth factor receptor substrate 2	1.31	8.66E-03	0.16
AASDH	aminoadipate-semialdehyde dehydrogenase	1.31	7.55E-03	0.16
PTPRE	protein tyrosine phosphatase, receptor type E	1.30	8.28E-03	0.16
PIKFYVE	phosphoinositide kinase, FYVE-type zinc finger containing	1.30	5.78E-03	0.15
PLCB1	phospholipase C beta 1	1.30	7.41E-03	0.16
ARPC4-TTLL3	ARPC4-TTLL3 readthrough	1.30	9.61E-03	0.16
CSGALNACT1	chondroitin sulfate N-acetylgalactosaminyltransferase 1	1.30	8.73E-03	0.16
ATF7IP	activating transcription factor 7 interacting protein	1.29	5.05E-03	0.15
LRP11	LDL receptor related protein 11	1.29	4.16E-03	0.14
CCNI	cyclin I	1.29	6.68E-03	0.15
JADE3	jade family PHD finger 3	1.29	2.61E-03	0.14
PHLDB2	pleckstrin homology like domain family B member 2	1.28	8.90E-03	0.16
PHIP	pleckstrin homology domain interacting protein	1.28	2.14E-03	0.13
FBXL4	F-box and leucine rich repeat protein 4	1.28	8.70E-04	0.10
CDC42BPA	CDC42 binding protein kinase alpha	1.28	4.51E-03	0.14
COL14A1	collagen type XIV alpha 1 chain	1.28	1.34E-03	0.12
AGO4	argonaute 4, RISC catalytic component	1.28	3.86E-04	0.08
LRRC58	leucine rich repeat containing 58	1.27	9.27E-04	0.10
EDNRB	endothelin receptor type B	1.27	6.92E-03	0.16
NEO1	neogenin 1	1.27	4.06E-03	0.14
FAM129A	family with sequence similarity 129 member A	1.27	8.57E-03	0.16
R3HDM2	R3H domain containing 2	1.26	9.06E-04	0.10
SP110	SP110 nuclear body protein	1.26	3.34E-03	0.14
RCBTB2	RCC1 and BTB domain containing protein 2	1.26	3.53E-03	0.14
IRF2	interferon regulatory factor 2	1.26	8.62E-03	0.16
ZNF846	zinc finger protein 846	1.26	8.46E-03	0.16
ETS1	ETS proto-oncogene 1, transcription factor	1.26	3.02E-03	0.14
CARF	calcium responsive transcription factor	1.25	7.79E-04	0.10
LY9	lymphocyte antigen 9	1.25	8.58E-03	0.16

SETD5	SET domain containing 5	1.24	3.62E-03	0.14
ELF1	E74 like ETS transcription factor 1	1.22	4.18E-03	0.14
EMSY	EMSY, BRCA2 interacting transcriptional repressor	1.20	9.09E-03	0.16
COPG2	coatomer protein complex subunit gamma 2	1.19	7.90E-03	0.16
KMT2A	lysine methyltransferase 2A	1.19	9.98E-03	0.16
SRP68	signal recognition particle 68	-1.16	6.64E-03	0.15
EIF2AK1	eukaryotic translation initiation factor 2 alpha kinase 1	-1.16	7.23E-03	0.16
SF3A3	splicing factor 3a subunit 3	-1.16	2.49E-03	0.14
PTBP1	polypyrimidine tract binding protein 1	-1.17	1.62E-03	0.12
CUL4A	cullin 4A	-1.17	8.98E-03	0.16
DNAJB12	DnaJ heat shock protein family (Hsp40) member B12	-1.17	5.37E-03	0.15
ACTN1	actinin alpha 1	-1.18	8.57E-03	0.16
BOLA3	bolA family member 3	-1.18	9.84E-03	0.16
RBFA	ribosome binding factor A (putative)	-1.19	7.34E-03	0.16
RUNDC1	RUN domain containing 1	-1.19	5.91E-03	0.15
HDGF	heparin binding growth factor	-1.19	8.47E-03	0.16
CD59	CD59 molecule (CD59 blood group)	-1.19	1.05E-03	0.11
PI4K2A	phosphatidylinositol 4-kinase type 2 alpha	-1.20	4.03E-03	0.14
RBM14-RBM4	RBM14-RBM4 readthrough	-1.20	7.05E-03	0.16
RAD23A	RAD23 homolog A, nucleotide excision repair protein	-1.20	4.90E-03	0.14
EIF3D	eukaryotic translation initiation factor 3 subunit D	-1.20	6.43E-03	0.15
CNOT9	CCR4-NOT transcription complex subunit 9	-1.20	5.94E-03	0.15
CHCHD1	coiled-coil-helix-coiled-coil-helix domain containing 1	-1.20	8.96E-03	0.16
BPNT1	3'(2'), 5'-bisphosphate nucleotidase 1	-1.20	1.65E-03	0.12
KRI1	KRI1 homolog	-1.20	8.95E-03	0.16
DEGS1	delta 4-desaturase, sphingolipid 1	-1.20	9.61E-03	0.16
MRM3	mitochondrial rRNA methyltransferase 3	-1.20	6.50E-03	0.15
NMT1	N-myristoyltransferase 1	-1.21	3.37E-03	0.14
CHFR	checkpoint with forkhead and ring finger domains	-1.21	4.78E-03	0.14
VAMP3	vesicle associated membrane protein 3	-1.21	2.18E-03	0.13
FAM136A	family with sequence similarity 136 member A	-1.21	3.55E-03	0.14
SLBP	stem-loop binding protein	-1.21	8.18E-03	0.16
RNPS1	RNA binding protein with serine rich domain 1	-1.21	7.03E-03	0.16
YARS	tyrosyl-tRNA synthetase	-1.21	4.80E-03	0.14
RCC2	regulator of chromosome condensation 2	-1.21	4.30E-03	0.14
TXLNA	taxilin alpha	-1.21	3.95E-03	0.14
MESDC2	mesoderm development candidate 2	-1.22	5.84E-03	0.15
DNAJC22	DnaJ heat shock protein family (Hsp40) member C22	-1.22	9.91E-03	0.16
PITPNA	phosphatidylinositol transfer protein alpha	-1.22	6.00E-03	0.15
EIF4G1	eukaryotic translation initiation factor 4 gamma 1	-1.22	7.65E-03	0.16
USP39	ubiquitin specific peptidase 39	-1.22	2.66E-03	0.14
POP4	POP4 homolog, ribonuclease P/MRP subunit	-1.22	9.56E-03	0.16
TIMM23	translocase of inner mitochondrial membrane 23	-1.22	8.89E-03	0.16
SUPT5H	SPT5 homolog, DSIF elongation factor subunit	-1.22	8.54E-03	0.16
VPS4A	vacuolar protein sorting 4 homolog A	-1.23	8.04E-03	0.16
GATAD2A	GATA zinc finger domain containing 2A	-1.23	9.77E-03	0.16
DTNB	dystrobrevin beta	-1.23	7.63E-03	0.16
SNU13	small nuclear ribonucleoprotein 13	-1.23	2.59E-03	0.14
DDX23	DEAD-box helicase 23	-1.23	7.74E-03	0.16
VPS72	vacuolar protein sorting 72 homolog	-1.23	2.94E-03	0.14
POLR1B	RNA polymerase I subunit B	-1.23	1.36E-03	0.12
PSMD4	proteasome 26S subunit, non-ATPase 4	-1.23	2.59E-03	0.14
DNAJC16	DnaJ heat shock protein family (Hsp40) member C16	-1.23	2.39E-03	0.13
BABAM2	BRISC and BRCA1 A complex member 2	-1.23	9.40E-03	0.16
DNAJC11	DnaJ heat shock protein family (Hsp40) member C11	-1.23	1.75E-03	0.12
AARS	alanyl-tRNA synthetase	-1.23	5.88E-03	0.15

SRPRB	SRP receptor beta subunit	-1.23	2.18E-03	0.13
CCT5	chaperonin containing TCP1 subunit 5	-1.23	5.27E-04	0.08
PSMD13	proteasome 26S subunit, non-ATPase 13	-1.23	6.53E-03	0.15
CCT7	chaperonin containing TCP1 subunit 7	-1.23	7.82E-03	0.16
EDF1	endothelial differentiation related factor 1	-1.24	6.17E-03	0.15
TMEM129	transmembrane protein 129	-1.24	8.46E-03	0.16
DRAP1	DR1 associated protein 1	-1.24	9.48E-03	0.16
DHX8	DEAH-box helicase 8	-1.24	6.92E-04	0.10
GBA2	glucosylceramidase beta 2	-1.24	4.82E-03	0.14
RPL7L1	ribosomal protein L7 like 1	-1.24	3.99E-03	0.14
MARK2	microtubule affinity regulating kinase 2	-1.24	9.08E-03	0.16
CCT3	chaperonin containing TCP1 subunit 3	-1.24	1.14E-03	0.11
SNRPB	small nuclear ribonucleoprotein polypeptides B and B1	-1.24	3.27E-03	0.14
GNA11	G protein subunit alpha 11	-1.24	2.59E-03	0.14
STK11	serine/threonine kinase 11	-1.24	7.25E-03	0.16
DNAJC17	DnaJ heat shock protein family (Hsp40) member C17	-1.24	5.31E-03	0.15
GRB10	growth factor receptor bound protein 10	-1.24	3.17E-03	0.14
ACLY	ATP citrate lyase	-1.24	2.41E-03	0.13
TMEM259	transmembrane protein 259	-1.24	7.66E-03	0.16
SH3BP5L	SH3 binding domain protein 5 like	-1.25	7.25E-04	0.10
UBC	ubiquitin C	-1.25	9.31E-03	0.16
BRPF1	bromodomain and PHD finger containing 1	-1.25	5.07E-03	0.15
FTSJ1	FtsJ RNA methyltransferase homolog 1 (E. coli)	-1.25	6.31E-03	0.15
RITA1	RBPJ interacting and tubulin associated 1	-1.26	4.34E-03	0.14
GDF2	growth differentiation factor 2	-1.26	3.22E-03	0.14
EARS2	glutamyl-tRNA synthetase 2, mitochondrial	-1.26	1.12E-03	0.11
ADPGK	ADP dependent glucokinase	-1.26	7.88E-03	0.16
DDX47	DEAD-box helicase 47	-1.26	1.68E-04	0.07
SDF2	stromal cell derived factor 2	-1.26	2.23E-03	0.13
TTC4	tetratricopeptide repeat domain 4	-1.26	9.33E-03	0.16
C17orf62	chromosome 17 open reading frame 62	-1.26	7.55E-03	0.16
ACO2	aconitase 2	-1.26	9.59E-03	0.16
PSMD8	proteasome 26S subunit, non-ATPase 8	-1.26	5.64E-03	0.15
NDUFB10	NADH:ubiquinone oxidoreductase subunit B10	-1.26	5.74E-03	0.15
NR2F6	nuclear receptor subfamily 2 group F member 6	-1.26	7.83E-03	0.16
SF3B4	splicing factor 3b subunit 4	-1.26	4.44E-03	0.14
NABP2	nucleic acid binding protein 2	-1.27	1.79E-03	0.13
GPATCH3	G-patch domain containing 3	-1.27	3.53E-03	0.14
ASGR1	asialoglycoprotein receptor 1	-1.27	5.18E-03	0.15
MS4A6A	membrane spanning 4-domains A6A	-1.27	6.61E-03	0.15
KNOP1	lysine rich nucleolar protein 1	-1.27	7.45E-03	0.16
DRG1	developmentally regulated GTP binding protein 1	-1.27	3.00E-03	0.14
PPM1G	protein phosphatase, Mg2+/Mn2+ dependent 1G	-1.27	6.14E-03	0.15
RBM19	RNA binding motif protein 19	-1.27	6.10E-03	0.15
NPLOC4	NPL4 homolog, ubiquitin recognition factor	-1.27	6.31E-03	0.15
ORAI1	ORAI calcium release-activated calcium modulator 1	-1.27	2.38E-03	0.13
ZFYVE27	zinc finger FYVE-type containing 27	-1.27	6.53E-03	0.15
CHST15	carbohydrate sulfotransferase 15	-1.28	8.66E-03	0.16
EI24	EI24, autophagy associated transmembrane protein	-1.28	2.00E-03	0.13
DHX30	DExH-box helicase 30	-1.28	1.09E-03	0.11
PES1	pescadillo ribosomal biogenesis factor 1	-1.28	2.74E-04	0.07
PSMB6	proteasome subunit beta 6	-1.28	6.80E-03	0.16
NAA10	N(alpha)-acetyltransferase 10, NatA catalytic subunit	-1.28	9.59E-03	0.16
NFKBIB	NFKB inhibitor beta	-1.28	3.63E-03	0.14
TANGO2	transport and golgi organization 2 homolog	-1.28	7.03E-03	0.16

DHX35	DEAH-box helicase 35	-1.28	5.99E-03	0.15
TIMM22	translocase of inner mitochondrial membrane 22	-1.28	5.79E-03	0.15
TIGAR	TP53 induced glycolysis regulatory phosphatase	-1.28	3.34E-03	0.14
POLR1A	RNA polymerase I subunit A	-1.28	1.88E-03	0.13
BCKDK	branched chain ketoacid dehydrogenase kinase	-1.28	4.40E-03	0.14
AAR2	AAR2 splicing factor homolog	-1.28	9.66E-03	0.16
NOP2	NOP2 nucleolar protein	-1.28	5.43E-03	0.15
TIMM13	translocase of inner mitochondrial membrane 13	-1.29	3.86E-04	0.08
BIN3	bridging integrator 3	-1.29	8.75E-03	0.16
MIF	macrophage migration inhibitory factor (glycosylation-inhibiting factor)	-1.29	8.62E-03	0.16
EMC1	ER membrane protein complex subunit 1	-1.29	5.80E-03	0.15
MRPL43	mitochondrial ribosomal protein L43	-1.29	1.24E-03	0.11
RNF25	ring finger protein 25	-1.29	7.48E-03	0.16
ACTR5	ARP5 actin-related protein 5 homolog	-1.29	6.98E-03	0.16
DDC	dopa decarboxylase	-1.29	3.47E-03	0.14
CDK2AP2	cyclin dependent kinase 2 associated protein 2	-1.29	4.13E-03	0.14
TBC1D8	TBC1 domain family member 8	-1.30	1.00E-03	0.11
B3GAT3	beta-1,3-glucuronyltransferase 3	-1.30	3.47E-03	0.14
PITRM1	pitrilysin metallopeptidase 1	-1.30	2.25E-03	0.13
MRPL20	mitochondrial ribosomal protein L20	-1.30	5.06E-03	0.15
MFSD4B	major facilitator superfamily domain containing 4B	-1.30	6.10E-03	0.15
FBXO31	F-box protein 31	-1.30	4.83E-03	0.14
MYH9	myosin heavy chain 9	-1.30	8.86E-03	0.16
CMTR1	cap methyltransferase 1	-1.30	9.24E-03	0.16
PAM16	presequence translocase associated motor 16 homolog	-1.30	8.99E-03	0.16
JOSD2	Josephin domain containing 2	-1.30	5.41E-03	0.15
PSME3	proteasome activator subunit 3	-1.30	6.39E-04	0.09
ALG9	ALG9, alpha-1,2-mannosyltransferase	-1.30	2.39E-03	0.13
SMCR8	Smith-Magenis syndrome chromosome region, candidate 8	-1.30	7.10E-03	0.16
MFSD5	major facilitator superfamily domain containing 5	-1.30	8.01E-03	0.16
SMARCB1	SWI/SNF related, matrix associated, actin dependent regulator of chromatin, subfamily b, member 1	-1.30	5.47E-03	0.15
PRMT1	protein arginine methyltransferase 1	-1.30	4.84E-03	0.14
POLR2H	RNA polymerase II subunit H	-1.30	5.16E-03	0.15
RANGAP1	Ran GTPase activating protein 1	-1.30	9.72E-03	0.16
COG8	component of oligomeric golgi complex 8	-1.30	8.32E-03	0.16
RABAC1	Rab acceptor 1	-1.30	8.88E-03	0.16
MMACHC	methylmalonic aciduria (cobalamin deficiency) cblC type, with homocystinuria	-1.30	3.77E-03	0.14
SAP30BP	SAP30 binding protein	-1.31	3.30E-03	0.14
MUL1	mitochondrial E3 ubiquitin protein ligase 1	-1.31	3.62E-03	0.14
ILF2	interleukin enhancer binding factor 2	-1.31	7.77E-04	0.10
C16orf91	chromosome 16 open reading frame 91	-1.31	6.85E-03	0.16
XPO5	exportin 5	-1.31	2.74E-03	0.14
CHCHD10	coiled-coil-helix-coiled-coil-helix domain containing 10	-1.31	7.23E-03	0.16
IMP3	IMP3, U3 small nucleolar ribonucleoprotein	-1.31	5.24E-03	0.15
TFPT	TCF3 fusion partner	-1.31	6.89E-04	0.10
MRPS11	mitochondrial ribosomal protein S11	-1.31	9.52E-03	0.16
UBL4A	ubiquitin like 4A	-1.31	4.55E-03	0.14
GSK3A	glycogen synthase kinase 3 alpha	-1.31	6.03E-03	0.15
HMBS	hydroxymethylbilane synthase	-1.31	1.56E-03	0.12
MIDN	midnolin	-1.31	4.16E-03	0.14
DDX54	DEAD-box helicase 54	-1.31	3.77E-03	0.14
GDPGP1	GDP-D-glucose phosphorylase 1	-1.31	7.51E-03	0.16

DHX37	DEAH-box helicase 37	-1.31	4.89E-03	0.14
RABIF	RAB interacting factor	-1.31	4.55E-05	0.05
TGFBRAP1	transforming growth factor beta receptor associated protein 1	-1.32	8.93E-03	0.16
VSIR	V-set immunoregulatory receptor	-1.32	7.83E-03	0.16
P4HA2	prolyl 4-hydroxylase subunit alpha 2	-1.32	1.23E-03	0.11
WDR46	WD repeat domain 46	-1.32	3.45E-03	0.14
METTL1	methyltransferase like 1	-1.32	1.54E-03	0.12
PFN1	profilin 1	-1.32	5.75E-03	0.15
DET1	de-etiolated homolog 1 (Arabidopsis)	-1.32	5.72E-03	0.15
CACTIN	cactin, spliceosome C complex subunit	-1.32	3.25E-03	0.14
YWHAH	tyrosine 3-monooxygenase/tryptophan 5-monooxygenase activation protein eta	-1.32	3.00E-03	0.14
ITPKC	inositol-trisphosphate 3-kinase C	-1.32	8.02E-03	0.16
ACTR1A	ARP1 actin-related protein 1 homolog A, centractin alpha	-1.32	2.22E-04	0.07
EIF4A1	eukaryotic translation initiation factor 4A1	-1.32	1.11E-03	0.11
HNRNPAB	heterogeneous nuclear ribonucleoprotein A/B	-1.33	8.72E-04	0.10
YIPF1	Yip1 domain family member 1	-1.33	7.65E-03	0.16
MFSD9	major facilitator superfamily domain containing 9	-1.33	5.45E-03	0.15
FKBP4	FK506 binding protein 4	-1.33	2.68E-04	0.07
DDX49	DEAD-box helicase 49	-1.33	4.35E-03	0.14
ODC1	ornithine decarboxylase 1	-1.33	2.40E-03	0.13
DDX27	DEAD-box helicase 27	-1.33	6.76E-03	0.16
HARS	histidyl-tRNA synthetase	-1.33	2.38E-03	0.13
NOP9	NOP9 nucleolar protein	-1.33	3.82E-03	0.14
FOLR1	folate receptor 1	-1.33	9.61E-03	0.16
TOE1	target of EGR1, member 1 (nuclear)	-1.33	1.15E-03	0.11
BYSL	bystin like	-1.33	4.78E-03	0.14
MBD3	methyl-CpG binding domain protein 3	-1.34	3.28E-03	0.14
LIN37	lin-37 DREAM MuvB core complex component	-1.34	2.19E-03	0.13
ERCC1	ERCC excision repair 1, endonuclease non-catalytic subunit	-1.34	5.12E-03	0.15
TPI1	triosephosphate isomerase 1	-1.34	6.12E-03	0.15
CAMK1	calcium/calmodulin dependent protein kinase I	-1.34	5.56E-03	0.15
APBA3	amyloid beta precursor protein binding family A member 3	-1.34	9.47E-04	0.10
AKIP1	A-kinase interacting protein 1	-1.34	4.06E-03	0.14
RRP1B	ribosomal RNA processing 1B	-1.35	1.51E-03	0.12
ZNF48	zinc finger protein 48	-1.35	8.11E-03	0.16
ZNF32	zinc finger protein 32	-1.35	9.33E-04	0.10
HCCS	holocytochrome c synthase	-1.35	5.55E-03	0.15
PGP	phosphoglycolate phosphatase	-1.35	2.69E-03	0.14
MAN1B1	mannosidase alpha class 1B member 1	-1.35	5.34E-03	0.15
OPLAH	5-oxoprolinase (ATP-hydrolysing)	-1.35	2.57E-03	0.14
FEN1	flap structure-specific endonuclease 1	-1.35	1.85E-03	0.13
TP53RK	TP53 regulating kinase	-1.35	1.28E-03	0.12
TMEM51	transmembrane protein 51	-1.35	4.57E-03	0.14
PHKG2	phosphorylase kinase catalytic subunit gamma 2	-1.35	4.60E-03	0.14
TOMM40	translocase of outer mitochondrial membrane 40	-1.35	3.59E-03	0.14
KTI12	KTI12 chromatin associated homolog	-1.35	1.04E-03	0.11
ELAC2	elaC ribonuclease Z 2	-1.35	7.65E-03	0.16
USP5	ubiquitin specific peptidase 5	-1.35	6.44E-03	0.15
SLC25A22	solute carrier family 25 member 22	-1.36	4.42E-03	0.14
DOHH	deoxyhypusine hydroxylase	-1.36	7.88E-04	0.10
FTSJ3	FtsJ homolog 3	-1.36	7.09E-04	0.10
KBTBD4	kelch repeat and BTB domain containing 4	-1.36	2.35E-03	0.13

AIMP2	aminoacyl tRNA synthetase complex interacting multifunctional protein 2	-1.36	4.77E-03	0.14
MLEC	malectin	-1.36	4.50E-03	0.14
SLC25A38	solute carrier family 25 member 38	-1.36	9.37E-03	0.16
TMCO6	transmembrane and coiled-coil domains 6	-1.36	9.21E-03	0.16
TINAGL1	tubulointerstitial nephritis antigen like 1	-1.36	2.50E-03	0.14
KCTD5	potassium channel tetramerization domain containing 5	-1.36	9.62E-03	0.16
SEC14L1	SEC14 like lipid binding 1	-1.36	6.45E-03	0.15
EXOSC5	exosome component 5	-1.36	4.41E-03	0.14
PPP1R14B	protein phosphatase 1 regulatory inhibitor subunit 14B	-1.37	5.21E-03	0.15
NAGS	N-acetylglutamate synthase	-1.37	8.21E-03	0.16
FARSA	phenylalanyl-tRNA synthetase alpha subunit	-1.37	3.84E-03	0.14
UTP4	UTP4, small subunit processome component	-1.37	9.77E-05	0.06
ABCA1	ATP binding cassette subfamily A member 1	-1.37	2.74E-03	0.14
EIF4EBP1	eukaryotic translation initiation factor 4E binding protein 1	-1.37	4.38E-03	0.14
CHPF	chondroitin polymerizing factor	-1.37	5.13E-03	0.15
ANXA2	annexin A2	-1.37	9.31E-03	0.16
IFNLR1	interferon lambda receptor 1	-1.38	9.28E-03	0.16
PELI3	pellino E3 ubiquitin protein ligase family member 3	-1.38	1.25E-03	0.11
TUBG1	tubulin gamma 1	-1.38	1.36E-03	0.12
GET4	golgi to ER traffic protein 4	-1.38	7.43E-03	0.16
PRMT5	protein arginine methyltransferase 5	-1.38	2.99E-04	0.07
NUP210	nucleoporin 210	-1.38	4.71E-03	0.14
RHBDD2	rhomboid domain containing 2	-1.38	7.29E-03	0.16
CARD19	caspase recruitment domain family member 19	-1.38	2.10E-03	0.13
JPT2	Jupiter microtubule associated homolog 2	-1.38	3.14E-03	0.14
E2F4	E2F transcription factor 4	-1.38	2.59E-05	0.04
TUBB4B	tubulin beta 4B class IVb	-1.39	3.36E-03	0.14
CHCHD4	coiled-coil-helix-coiled-coil-helix domain containing 4	-1.39	1.12E-03	0.11
TUBA1B	tubulin alpha 1b	-1.39	8.50E-03	0.16
SGMS1	sphingomyelin synthase 1	-1.39	6.94E-03	0.16
CIAPIN1	cytokine induced apoptosis inhibitor 1	-1.39	2.11E-03	0.13
OAF	out at first homolog	-1.39	2.54E-03	0.14
RRP9	ribosomal RNA processing 9, small subunit (SSU) processome component, homolog (yeast)	-1.39	5.12E-03	0.15
SLC23A1	solute carrier family 23 member 1	-1.39	2.55E-03	0.14
REXO4	REX4 homolog, 3'-5' exonuclease	-1.40	9.69E-04	0.11
KAT2A	lysine acetyltransferase 2A	-1.40	2.42E-04	0.07
SYTL4	synaptotagmin like 4	-1.40	1.62E-03	0.12
FADD	Fas associated via death domain	-1.40	5.61E-03	0.15
MON1A	MON1 homolog A, secretory trafficking associated	-1.40	7.44E-04	0.10
ABCF2	ATP binding cassette subfamily F member 2	-1.40	6.32E-05	0.05
CCND1	cyclin D1	-1.40	1.65E-03	0.12
DDX28	DEAD-box helicase 28	-1.41	1.44E-03	0.12
PEMT	phosphatidylethanolamine N-methyltransferase	-1.41	7.80E-04	0.10
VAC14	Vac14, PIKFYVE complex component	-1.41	5.11E-03	0.15
WDR74	WD repeat domain 74	-1.41	2.85E-04	0.07
MARS	methionyl-tRNA synthetase	-1.41	6.71E-03	0.15
SERTAD3	SERTA domain containing 3	-1.41	8.36E-03	0.16
WDR77	WD repeat domain 77	-1.41	6.30E-05	0.05
WDR37	WD repeat domain 37	-1.42	9.48E-03	0.16
DNASE1L1	deoxyribonuclease 1 like 1	-1.42	4.84E-03	0.14
ZNHIT2	zinc finger HIT-type containing 2	-1.42	5.91E-03	0.15
RRP8	ribosomal RNA processing 8, methyltransferase, homolog (yeast)	-1.42	5.79E-03	0.15

PREP	prolyl endopeptidase	-1.42	3.23E-03	0.14
CD3EAP	CD3e molecule associated protein	-1.42	5.54E-03	0.15
HNF4A	hepatocyte nuclear factor 4 alpha	-1.42	4.44E-03	0.14
C20orf27	chromosome 20 open reading frame 27	-1.42	5.57E-03	0.15
NACC1	nucleus accumbens associated 1	-1.43	1.41E-03	0.12
PREB	prolactin regulatory element binding	-1.43	9.13E-04	0.10
H2AFX	H2A histone family member X	-1.43	3.49E-03	0.14
NOB1	NIN1/PSMD8 binding protein 1 homolog	-1.43	7.98E-03	0.16
NPR1	natriuretic peptide receptor 1	-1.43	3.88E-03	0.14
GAS2L1	growth arrest specific 2 like 1	-1.44	3.15E-04	0.07
URB2	URB2 ribosome biogenesis 2 homolog (S. cerevisiae)	-1.45	3.95E-04	0.08
NTMT1	N-terminal Xaa-Pro-Lys N-methyltransferase 1	-1.45	2.49E-04	0.07
AVPI1	arginine vasopressin induced 1	-1.45	6.27E-03	0.15
ERN1	endoplasmic reticulum to nucleus signaling 1	-1.45	4.89E-03	0.14
GNAZ	G protein subunit alpha z	-1.46	9.48E-04	0.10
POMGNT2	protein O-linked mannose N-acetylglucosaminyltransferase 2 (beta 1,4-)	-1.46	4.37E-03	0.14
FAM20C	FAM20C, golgi associated secretory pathway kinase	-1.46	7.27E-04	0.10
SELL	selectin L	-1.46	7.91E-03	0.16
SHH	sonic hedgehog	-1.46	6.27E-03	0.15
PPAN	peter pan homolog (Drosophila)	-1.46	3.37E-03	0.14
CCDC86	coiled-coil domain containing 86	-1.46	1.29E-05	0.03
P2RX4	purinergic receptor P2X 4	-1.46	1.21E-03	0.11
GRWD1	glutamate rich WD repeat containing 1	-1.47	5.03E-04	0.08
GLUL	glutamate-ammonia ligase	-1.47	9.45E-04	0.10
BCAR1	BCAR1, Cas family scaffolding protein	-1.47	1.81E-03	0.13
RRP12	ribosomal RNA processing 12 homolog	-1.48	1.44E-03	0.12
NR2C2AP	nuclear receptor 2C2 associated protein	-1.48	1.92E-04	0.07
PPRC1	peroxisome proliferator-activated receptor gamma, coactivator-related 1	-1.49	2.27E-03	0.13
TUBB	tubulin beta class I	-1.50	6.90E-03	0.16
LMNB2	lamin B2	-1.50	7.84E-06	0.02
NOL6	nucleolar protein 6	-1.50	1.48E-03	0.12
CYP4A22	cytochrome P450 family 4 subfamily A member 22	-1.50	8.85E-03	0.16
ADRA2B	adrenoceptor alpha 2B	-1.51	6.81E-04	0.10
TNFAIP1	TNF alpha induced protein 1	-1.51	8.98E-04	0.10
DPP3	dipeptidyl peptidase 3	-1.51	4.10E-03	0.14
SEMA3F	semaphorin 3F	-1.52	8.50E-03	0.16
SERTAD1	SERTA domain containing 1	-1.52	8.92E-03	0.16
GPRIN3	GPRIN family member 3	-1.52	4.87E-03	0.14
GIPC1	GIPC PDZ domain containing family member 1	-1.52	7.00E-03	0.16
PCTP	phosphatidylcholine transfer protein	-1.52	1.86E-03	0.13
RPUSD3	RNA pseudouridylate synthase domain containing 3	-1.52	1.24E-03	0.11
MEPCE	methylphosphate capping enzyme	-1.52	6.69E-03	0.15
CARD10	caspase recruitment domain family member 10	-1.53	1.04E-03	0.11
SDC1	syndecan 1	-1.53	2.26E-04	0.07
WDR4	WD repeat domain 4	-1.53	2.08E-03	0.13
DAGLB	diacylglycerol lipase beta	-1.53	2.98E-03	0.14
SLC2A1	solute carrier family 2 member 1	-1.54	7.48E-04	0.10
PLXNA1	plexin A1	-1.54	6.26E-03	0.15
NPM3	nucleophosmin/nucleoplasmin 3	-1.55	9.72E-03	0.16
ZNRD1	zinc ribbon domain containing 1	-1.56	3.56E-04	0.07
FOXA3	forkhead box A3	-1.56	1.16E-03	0.11
PPM1J	protein phosphatase, Mg2+/Mn2+ dependent 1J	-1.56	5.64E-03	0.15
TAGLN2	transgelin 2	-1.60	5.29E-06	0.02
CHAF1B	chromatin assembly factor 1 subunit B	-1.61	5.46E-03	0.15

NOD1	nucleotide binding oligomerization domain containing 1	-1.61	2.38E-03	0.13
FKBP1B	FK506 binding protein 1B	-1.61	6.35E-03	0.15
BANP	BTG3 associated nuclear protein	-1.61	3.04E-03	0.14
SLC12A4	solute carrier family 12 member 4	-1.62	4.75E-03	0.14
AMPD3	adenosine monophosphate deaminase 3	-1.62	9.91E-03	0.16
SRM	spermidine synthase	-1.64	2.77E-03	0.14
MCM3	minichromosome maintenance complex component 3	-1.65	2.37E-03	0.13
ITPR3	inositol 1,4,5-trisphosphate receptor type 3	-1.65	5.91E-03	0.15
MCM2	minichromosome maintenance complex component 2	-1.65	4.51E-03	0.14
PUDP	pseudouridine 5'-phosphatase	-1.66	2.33E-04	0.07
CKAP4	cytoskeleton associated protein 4	-1.67	3.70E-03	0.14
PTPMT1	protein tyrosine phosphatase, mitochondrial 1	-1.67	3.31E-05	0.04
TTLL4	tubulin tyrosine ligase like 4	-1.69	4.93E-04	0.08
CDCA5	cell division cycle associated 5	-1.70	7.87E-03	0.16
CTPS1	CTP synthase 1	-1.73	3.63E-03	0.14
ALOX5AP	arachidonate 5-lipoxygenase activating protein	-1.73	2.20E-04	0.07
SLC2A8	solute carrier family 2 member 8	-1.74	7.37E-03	0.16
E2F1	E2F transcription factor 1	-1.75	9.76E-03	0.16
DAPK2	death associated protein kinase 2	-1.83	1.77E-03	0.13
CRB3	crumbs 3, cell polarity complex component	-1.85	2.26E-03	0.13
KRT18	keratin 18	-1.86	2.93E-03	0.14
FAM83F	family with sequence similarity 83 member F	-1.88	4.85E-03	0.14
ELF3	E74 like ETS transcription factor 3	-1.89	6.25E-03	0.15
TTPAL	alpha tocopherol transfer protein like	-1.90	2.62E-04	0.07
ALAS1	5'-aminolevulinate synthase 1	-1.92	4.94E-03	0.15
EIF1AD	eukaryotic translation initiation factor 1A domain containing	-1.97	2.72E-03	0.14
CSF1	colony stimulating factor 1	-1.99	1.17E-03	0.11
ABCA3	ATP binding cassette subfamily A member 3	-2.00	3.96E-03	0.14
CYC1	cytochrome c1	-2.01	8.02E-03	0.16
SERPINA3	serpin family A member 3	-2.01	1.98E-04	0.07
DOK7	docking protein 7	-2.17	6.31E-03	0.15
ADM2	adrenomedullin 2	-2.23	2.37E-03	0.13
KCNE4	potassium voltage-gated channel subfamily E regulatory subunit 4	-2.49	4.01E-03	0.14
TPD52L1	tumor protein D52 like 1	-2.74	1.97E-04	0.07
MYCN	MYCN proto-oncogene, bHLH transcription factor	-2.76	1.67E-03	0.12
SAA3P	serum amyloid A3 pseudogene	-3.00	4.54E-03	0.14
RAB11FIP5	RAB11 family interacting protein 5	-3.86	3.39E-05	0.04
CCNT2	cyclin T2	-5.40	8.63E-04	0.10
