# Supplementary material for: RNA-Seq of Liver From Pigs Divergent in Feed Efficiency Highlights Shifts in Macronutrient Metabolism, Hepatic Growth and Immune Response
Source: Front Genet. 2019 Feb 19;10:117. doi: 10.3389/fgene.2019.00117 (PMC6389832; doi:10.3389/fgene.2019.00117)
Supplement: Supplementary file 2 [file Data_Sheet_2.PDF]

Supplementary material

RNA-seq of liver from pigs divergent in feed efficiency highlights shifts in macronutrient metabolism, hepatic growth and immune response

Justyna Horodyska, Ruth M. Hamill*, Henry Reyer, Nares Trakooljul, Peadar G. Lawlor, Ursula M. McCormack and Klaus Wimmers

*Corresponding author

Table S2 Molecular and cellular functions significantly over-represented among differentially expressed genes.

Category	*P*-value range	Sub-categories*
Gene Expression	1.65E-09-9.62E-04	transactivation of RNA (1.26), activation of DNA endogenous promoter (1.21), transactivation (1.01), transcription of RNA (0.79), transcription (0.68), expression of RNA (0.48), transcription of DNA (0.44)
Cellular Movement	1.44E-08-3.15E-03	cell movement of tumor cell lines (**2.11**), cell spreading of tumor cell lines (1.90), cell movement of leukemia cell lines (1.44), migration of cells (1.41), invasion of tumor cells (1.23), cell movement (1.08), migration of neural crest cells (1.07), cell movement of neuroblastoma cell lines (0.93), invasion of cells (0.88), migration of breast cancer cell lines (0.88), cell movement of neurons (0.8), migration of embryonic cells (0.73), homing of cells (0.71), migration of neurons (0.67), migration of colorectal cancer cell lines (0.56), migration of cortical neurons (0.45), cell movement of embryonic cells (0.39), chemotaxis (0.34), cell movement of colorectal cancer cell lines (0.13), cell movement of breast cell lines (0.11), migration of endothelial cells (0.09), homing of leukocytes (0.03), migration of cancer cells (0), chemotaxis of phagocytes (0), cell movement of endothelial cells (-0.04), mobilization of hematopoietic progenitor cells (-0.06), migration of vascular endothelial cells (-0.07), invasion of lung cancer cell lines (-0.15), cell movement of tumor cells (-0.21), chemotaxis of leukocytes (-0.32), migration of tumor cells (-0.42), recruitment of myeloid cells (-0.71), cell movement of connective tissue cells (-0.81), cell movement of prostate cancer cells (-1.07), cell movement of fibroblasts (-1.24)

Cellular Development	7.31E-07-3.32E-03	differentiation of tumor cell lines (**2.31**), differentiation of epithelial cells (**2.13**), branching of cells (1.99), development of tumor cells (1), differentiation of epithelial tissue (0.87), leukopoiesis (0.69), differentiation of mononuclear leukocytes (0.58), proliferation of hematopoietic progenitor cells (0.52), proliferation of hematopoietic cells (0.21), proliferation of hepatocytes (0.16), endothelial cell development (0.06), proliferation of lung cancer cell lines (-0.01), epithelial-mesenchymal transition (-0.03), differentiation of embryonic tissue (-0.1), differentiation of neural stem cells (-0.31), cell proliferation of cervical cancer cell lines (-0.45), proliferation of neural stem cells (-0.48), development of epithelial cells (-0.49), proliferation of lymphocytes (-0.55), proliferation of blood cells (-0.66), proliferation of mononuclear leukocytes (-0.68), proliferation of immune cells (-0.81), differentiation of embryonic cell lines (-0.84), differentiation of muscle cell lines (-0.88), proliferation of embryonic stem cells (-0.93), proliferation of fibroblast cell lines (-1.03), proliferation of stem cells (-1.36), cell proliferation of tumor cell lines (-1.4), proliferation of embryonic cells (-1.45), cell proliferation of fibroblasts (-1.69), proliferation of muscle cells (-1.90)
Cellular Growth and Proliferation	7.31E-07-3.32E-03	colony formation of cervical cancer cell lines (1.73), stimulation of lymphocytes (1.39), colony formation of cells (1.19), development of tumor cells (1), contact growth inhibition of colorectal cancer cell lines (0.99), stimulation of mononuclear leukocytes (0.94), stimulation of lymphatic system cells (0.9), stimulation of leukocytes (0.84), leukopoiesis (0.69), differentiation of mononuclear leukocytes (0.58), proliferation of hematopoietic progenitor cells (0.52), proliferation of hematopoietic cells (0.21), proliferation of hepatocytes (0.16), accumulation of tumor cell lines (0.11), endothelial cell development (0.06), proliferation of lung cancer cell lines (-0.01), cell proliferation of cervical cancer cell lines (-0.45), proliferation of neural stem cells (-0.48), development of epithelial cells (-0.49), proliferation of lymphocytes (-0.55), cytostasis (-0.56), proliferation of blood cells (-0.66), proliferation of mononuclear leukocytes (-0.68), proliferation of lymphatic system cells (-0.72), proliferation of immune cells (-0.81), proliferation of embryonic stem cells (-0.93), proliferation of connective tissue cells (-1.00), proliferation of fibroblast cell lines (-1.03), proliferation of stem cells (-1.36), cell proliferation of tumor cell lines (-1.4), proliferation of embryonic cells (-1.45), cell proliferation of fibroblasts (-1.69), proliferation of muscle cells (-1.90)
Post-Translational Modification	8.7E-07-9.45E-04	phosphorylation of protein (0.30)

Cell Cycle	1.02E-06-3.31E-03	G2 phase of tumor cell lines (1.00), entry into interphase of leukocytes (1.00), G2 phase of cervical cancer cell lines (1.00), contact growth inhibition of colorectal cancer cell lines (0.99), ploidy (0.67), interphase of blood cells (0.62), interphase of leukocytes (0.62), interphase of epithelial cells (0.19), aneuploidy of cells (0.15), G1/S phase transition (0.07), interphase (0.01), interphase of tumor cell lines (0), G2/M phase (0), G2 phase (-0.1), ploidy of cells (-0.19), aneuploidy (-0.28), cell cycle progression (-0.34), cell cycle progression of tumor cell lines (-0.66), S phase of fibroblasts (-0.85), S phase (-1.26), S phase of fibroblast cell lines (-1.71), recombination of cells (-1.73), senescence of cells (**-2.90**)
Cell Death and Survival	1.27E-06-2.85E-03	cell viability of natural killer cells (**2.20**), cell death of cancer cells (1.66), necrosis of tumor (1.43), cell death of tumor cells (1.43), apoptosis of cancer cells (1.39), cell viability of fibroblasts (1.13), apoptosis of tumor cells (1.11), cell viability of tumor cell lines (1), cell viability (0.55), cell survival (0.46), cell viability of tumor cells (0.27), necrosis (0.15), cell death (0.03), cell death of tumor cell lines (-0.02), cell death of immune cells (-0.16), apoptosis of tumor cell lines (-0.21), cell death of hematopoietic cell lines (-0.44), apoptosis (-0.45), cell death of blood cells (-0.48), cell death of leukocyte cell lines (-0.53), apoptosis of mononuclear leukocytes (-0.83), cell death of B-lymphocyte derived cell lines (-0.86), apoptosis of leukocytes (-0.91), apoptosis of blood cells (-1.00), anoikis (-1.63)
Carbohydrate Metabolism	2.53E-06-1.71E-03	uptake of D-glucose (0.05), uptake of D-hexose (-0.04), conversion of phosphatidic acid (-0.06), uptake of monosaccharide (-0.22), conversion of carbohydrate (-0.39), uptake of carbohydrate (-0.66)
Molecular Transport	2.53E-06-3.24E-03	secretion of protein (1.00), concentration of triacylglycerol (0.83), accumulation of triacylglycerol (0.37), uptake of D-glucose (0.05), concentration of acylglycerol (0.02), uptake of D-hexose (-0.04)
Small Molecule Biochemistry	2.53E-06-3.31E-03	concentration of triacylglycerol (0.83), accumulation of triacylglycerol (0.37), conversion of phospholipid (0.28), uptake of D-glucose (0.05), concentration of acylglycerol (0.02), uptake of D-hexose (-0.04), conversion of phosphatidic acid (-0.06), conversion of lipid (-1), incorporation of palmitic acid (-1.41), incorporation of lipid (-1.92)
Cell Morphology	6.62E-06-3.32E-03	branching of cells (1.99), formation of cellular protrusions (1.90), cell spreading of tumor cell lines (1.9), sprouting (1.7), polarization of cells (1.41), electrical resistance of endothelial cells (1.07), shape change of tumor cell lines (0.79), shape change of endothelial cell lines (0.69), shape change of fibroblast cell lines (0.05)
DNA Replication, Recombination, and Repair	2.69E-05-1.14E-03	DNA replication (0.93), re-replication of DNA (0.28), metabolism of DNA (0.09), synthesis of DNA (-0.99), recombination of cells (-1.73)

Cell-To-Cell Signaling and Interaction	6.63E-05-3.32E-03	stimulation of lymphocytes (1.39), electrical resistance of endothelial cells (1.07), contact growth inhibition of colorectal cancer cell lines (0.99), stimulation of mononuclear leukocytes (0.94), stimulation of lymphatic system cells (0.9), stimulation of leukocytes (0.84), adhesion of lung cancer cell lines (0.28), recruitment of myeloid cells (-0.71)
Protein Synthesis	9.28E-05-8.64E-04	metabolism of protein (0.67), catabolism of protein (**2.16**)
Cellular Assembly and Organization	9.87E-05-3.32E-03	organization of cytoskeleton (**2.40**), organization of cytoplasm (**2.38**), microtubule dynamics (**2.23**), formation of cellular protrusions (1.90), secretion of vesicles (1.67), electrical resistance of endothelial cells (1.07), cohesion of sister chromatids (0.93), growth of microtubules (0.85)
Cellular Function and Maintenance	9.87E-05-2.66E-03	organization of cytoskeleton (**2.40**), organization of cytoplasm (**2.38)**, microtubule dynamics (**2.23**), formation of cellular protrusions (1.90), secretion of vesicles (1.67), growth of microtubules (0.85), endothelial cell development (0.06), cell saturation density (-0.76)
Lipid Metabolism	2.13E-04-3.31E-03	concentration of triacylglycerol (0.83), accumulation of triacylglycerol (0.37), conversion of phospholipid (0.28), concentration of acylglycerol (0.02), conversion of phosphatidic acid (-0.06), conversion of lipid (-1.00), incorporation of palmitic acid (-1.41), incorporation of lipid (-1.92)
RNA Post-Transcriptional Modification	3.87E-04-3.87E-04	processing of RNA (0.85)
Cell Signaling	4.99E-04-9.45E-04	stimulation of lymphocytes (1.39), electrical resistance of endothelial cells (1.07), contact growth inhibition of colorectal cancer cell lines (0.99), stimulation of mononuclear leukocytes (0.94), stimulation of lymphatic system cells (0.9), stimulation of leukocytes (0.84), adhesion of lung cancer cell lines (0.28), recruitment of myeloid cells (-0.71)
Protein Degradation	8.64E-04-8.64E-04	catabolism of protein (**2.16**)
Protein Trafficking	2.04E-03-2.04E-03	secretion of protein (1.00)

*Significantly activated (z-score > 2) sub-categories are highlighted in red and significantly inhibited sub-categories are highlighted in green (z-score < -2)

NA: no available z-score
