# Supplementary material for: RNA-Seq of Liver From Pigs Divergent in Feed Efficiency Highlights Shifts in Macronutrient Metabolism, Hepatic Growth and Immune Response
Source: Front Genet. 2019 Feb 19;10:117. doi: 10.3389/fgene.2019.00117 (PMC6389832; doi:10.3389/fgene.2019.00117)
Supplement: Supplementary file 3 [file Data_Sheet_3.PDF]

Supplementary material

RNA-seq of liver from pigs divergent in feed efficiency highlights shifts in macronutrient metabolism, hepatic growth and immune response

Justyna Horodyska, Ruth M. Hamill*, Henry Reyer, Nares Trakooljul, Peadar G. Lawlor, Ursula M. McCormack and Klaus Wimmers

**Corresponding author*

Table S3 Physiological system development and function categories significantly over-represented among differentially expressed genes.

Category	*P*-value range	Sub-categories*
Cardiovascular System Development and Function	1.44E-08-3.32E-03	cardiac contractility (1.56), vasculogenesis (1.1), electrical resistance of endothelial cells (1.07), angiogenesis (0.94), development of vasculature (0.94), cardiac output (0.47), migration of endothelial cells (0.09), development of endothelial tissue (0.06), endothelial cell development (0.06), cell movement of endothelial cells (-0.04), migration of vascular endothelial cells (-0.07), density of blood vessel (-0.61), function of heart ventricle (**-2.24**)
Organismal Survival	6.03E-07-2.10E-04	morbidity or mortality (-2.24), organismal death (-1.96), perinatal death (-1.29)
Connective Tissue Development and Function	5.33E-06-3.15E-03	cell viability of fibroblasts (1.13), shape change of fibroblast cell lines (0.05), S phase of fibroblasts (-0.85), proliferation of connective tissue cells (-1), proliferation of fibroblast cell lines (-1.03), growth of connective tissue (-1.24), cell movement of fibroblasts (-1.24), cell proliferation of fibroblasts (-1.69), S phase of fibroblast cell lines (-1.71)
Tissue Morphology	8.34E-06-3.32E-03	quantity of lymphocytes (**2.40**), quantity of invariant natural killer T cells (**2.10**), quantity of B lymphocytes (1.94), quantity of blood cells (1.88), quantity of stem cells (1.26), electrical resistance of endothelial cells (1.07), density of blood vessel (-0.61), quantity of hematopoietic progenitor cells (-0.84)
Organismal Development	8.81E-06-3.35E-03	vasculogenesis (1.1), angiogenesis (0.94), endothelial cell development (0.06), differentiation of embryonic tissue (-0.1), differentiation of neural stem cells (-0.31), proliferation of neural stem cells (-0.48), density of blood vessel (-0.61), proliferation of embryonic stem cells (-0.93), development of body trunk (-0.99), development of blastocyst (-1), growth of embryo (-1.36), proliferation of embryonic cells (-1.45), growth of organism (-1.65)

Tissue Development	1.44E-05-3.32E-03	differentiation of epithelial cells (**2.13**), accumulation of CD4+ T-lymphocytes (2.00), branching of epithelial tissue (1.35), cell viability of fibroblasts (1.13), differentiation of epithelial tissue (0.87), leukopoiesis (0.69), mineralization of extracellular matrix (0.65), differentiation of mononuclear leukocytes (0.58), proliferation of hepatocytes (0.16), development of endothelial tissue (0.06), endothelial cell development (0.06), shape change of fibroblast cell lines (0.05), differentiation of embryonic tissue (-0.1), development of epithelial tissue (-0.24), growth of epithelial tissue (-0.24), differentiation of neural stem cells (-0.31), proliferation of neural stem cells (-0.48), development of epithelial cells (-0.49), S phase of fibroblasts (-0.85), proliferation of connective tissue cells (-1.00), proliferation of fibroblast cell lines (-1.03), growth of connective tissue (-1.24), cell movement of fibroblasts (-1.24), cell proliferation of fibroblasts (-1.69), S phase of fibroblast cell lines (-1.71), proliferation of muscle cells (-1.9)
Hematological System Development and Function	1.61E-05-3.32E-03	quantity of lymphocytes (**2.40**), cell viability of natural killer cells (**2.20**), quantity of invariant natural killer T cells (**2.10**), accumulation of CD4+ T-lymphocytes (2), quantity of B lymphocytes (1.94), quantity of blood cells (1.88), stimulation of lymphocytes (1.39), stimulation of mononuclear leukocytes (0.94), stimulation of leukocytes (0.84), leukopoiesis (0.69), differentiation of mononuclear leukocytes (0.58), proliferation of hematopoietic progenitor cells (0.52), proliferation of hematopoietic cells (0.21), homing of leukocytes (0.03), chemotaxis of phagocytes (0), mobilization of hematopoietic progenitor cells (-0.06), chemotaxis of leukocytes (-0.32), proliferation of lymphocytes (-0.55), proliferation of mononuclear leukocytes (-0.68), proliferation of immune cells (-0.81), quantity of hematopoietic progenitor cells (-0.84)
Lymphoid Tissue Structure and Development	1.61E-05-3.32E-03	quantity of lymphocytes (**2.40**), quantity of invariant natural killer T cells (**2.10**), quantity of B lymphocytes (1.94), leukopoiesis (0.69), differentiation of mononuclear leukocytes (0.58), proliferation of lymphocytes (-0.55), proliferation of mononuclear leukocytes (-0.68), proliferation of lymphatic system cells (-0.72), proliferation of immune cells (-0.81)
Embryonic Development	4.22E-05-3.32E-03	migration of neural crest cells (1.07), migration of embryonic cells (0.73), cell movement of embryonic cells (0.39), quantity of embryo (0.2), differentiation of embryonic tissue (-0.1), differentiation of neural stem cells (-0.31), proliferation of neural stem cells (-0.48), differentiation of embryonic cell lines (-0.84), proliferation of embryonic stem cells (-0.93), development of body trunk (-0.99), development of blastocyst (-1.00), growth of embryo (-1.36), proliferation of embryonic cells (-1.45)
Organ Development	4.22E-05-3.32E-03	cardiac contractility (1.56), cardiac output (0.47), proliferation of hepatocytes (0.16), proliferation of muscle cells (-1.9), function of heart ventricle (**-2.24**)
Organ Morphology	4.22E-05-3.35E-03	cardiac contractility (1.56)
Respiratory System Development and Function	4.22E-05-7.88E-04	morphology of lung (NA), abnormal morphology of lung (NA), morphology of respiratory system (NA), formation of lung (NA), respiratory system development (NA)
Digestive System Development and Function	5.48E-05-3.35E-03	proliferation of hepatocytes (0.16)

Nervous System Development and Function	6.63E-05-2.52E-03	migration of neurons (0.68), migration of cortical neurons (0.45)
Skeletal and Muscular System Development and Function	1.50E-04-2.79E-03	differentiation of muscle cell lines (-0.88), proliferation of muscle cells (-1.90)
Hematopoiesis	2.67E-04-3.32E-03	proliferation of hematopoietic progenitor cells (0.52), proliferation of hematopoietic cells (0.21), leukopoiesis (0.69), mobilisation of hematopoietic progenitor cells (-0.06), differentiation of mononuclear leukocyte (0.58), quantity of hematopoietic progenitor cells (-0.84)
Immune Cell Trafficking	3.47E-04-1.44E-03	homing of leukocytes (0.03), accumulation of CD4+ T-lymphocytes (2.00), chemotaxis of leukocytes (-0.32), chemotaxis of phagocytes (-0.01)
Reproductive System Development and Function	1.06E-03-2.66E-03	cell movement of breast cell lines (0.13), cell cycle of germ cells (NA)
Hepatic System Development and Function	1.44E-03-3.35E-03	proliferation of hepatocytes (0.16)
Organismal Functions	1.44E-03-1.50E-03	inhibition of melanoma cells (NA), closure of wound (NA)
Humoral Immune Response	2.52E-03-3.32E-03	quantity of B lymphocytes (1.94), development of B-1 Lymphocytes (NA)

*Significantly activated (z-score > 2) sub-categories are highlighted in red and significantly inhibited sub-categories are highlighted in green (z-score < -2); NA: no available z-score
