# Supplementary material for: RNA-Seq of Liver From Pigs Divergent in Feed Efficiency Highlights Shifts in Macronutrient Metabolism, Hepatic Growth and Immune Response
Source: Front Genet. 2019 Feb 19;10:117. doi: 10.3389/fgene.2019.00117 (PMC6389832; doi:10.3389/fgene.2019.00117)
Supplement: Supplementary file 4 [file Data_Sheet_4.PDF]

*Supplementary material*

**RNA-seq of liver from pigs divergent in feed efficiency highlights shifts in macronutrient metabolism, hepatic growth and immune response**

Justyna Horodyska, Ruth M. Hamill*, Henry Reyer, Nares Trakooljul, Peadar G. Lawlor, Ursula M. McCormack and Klaus Wimmers

**Corresponding author*

**Table S4** Significantly enriched canonical signaling pathways identified in liver samples of feed efficiency divergent pigs.

| Ingenuity Canonical Pathways | -log(p-value) | z-score | Genes |
|---|---|---|---|
| Role of NFAT in Regulation of the Immune Response | 4.07 | 2.14 | *BLNK, AKAP5, GNG2, SOS2, GNA11, MEF2A, GSK3A, ITPR1, GNAZ, GAB1, SOS1, ITPR3, MS4A2, PLCB1, AKT3, MEF2C, NFKBIB, FRS2, ORAI1* |
| HGF Signaling | 3.36 | 2.33 | *ETS1, SOS2, MAP3K1, MAPK9, CCND1, ELF1, MET, ELF2, ELF3, GAB1, SOS1, AKT3, FRS2* |
| Aldosterone Signaling in Epithelial Cells | 3.21 | 2.12 | *DNAJC17, DNAJB12, DNAJB4, DNAJC6, SOS2, PIKFYVE, DNAJC13, ITPR1, DNAJC11, GAB1, SOS1, ITPR3, PLCB1, DNAJC22, FRS2, DNAJC16* |
| Gap Junction Signaling | 3.10 | NA | *TUBA1B, TUBB4B, SOS2, TUBG1, ITPR1, TUBB, PRKG1, GAB1, NPR1, ITPR3, SOS1, CAV1, PLCB1, AKT3, FRS2, GUCY1B3* |
| Cell Cycle Regulation by BTG Family Proteins | 2.73 | NA | *E2F4, PPM1J, E2F1, CCND1, PPP2R5A, PRMT1* |
| B Cell Receptor Signaling | 2.71 | 2.00 | *MAP2K6, ETS1, BLNK, SOS2, MAP3K1, MAPK9, GSK3A, PTEN, GAB1, CARD10, PAG1, SOS1, AKT3, MEF2C, NFKBIB, FRS2* |
| tRNA Charging | 2.48 | NA | *YARS, HARS, EARS2, AARS, MARS, FARSA* |
| ILK Signaling | 2.46 | 0.00 | *MAP2K6, MYH9, BMP2, MAPK9, GSK3A, RICTOR, CCND1, PPP1R14B, PPP2R5A, PTEN, GAB1, PPM1J, AKT3, KRT18, FRS2, ACTN1* |

| | | | |
|---|---|---|---|
| 14-3-3-mediated Signaling | 2.39 | 2.12 | *TUBA1B, YWHAH, GAB1, TUBB4B, STK11, TUBG1, MAPK9, AKT3, PLCB1, GSK3A, TUBB, FRS2* |
| EGF Signaling | 2.38 | 2.12 | *GAB1, SOS1, MAP3K1, ITPR3, SOS2, AKT3, ITPR1, FRS2* |
| Protein Ubiquitination Pathway | 2.38 | NA | *DNAJC17, DNAJB12, DNAJB4, USP5, PSMD13, DNAJC6, DNAJC13, PSMB6, USP39, PSMD8, DNAJC11, USP47, PSMD4, UBC, DNAJC22, USP34, DNAJC16, BIRC3, BIRC2* |
| EIF2 Signaling | 2.32 | 0.30 | *EIF2AK1, MYCN, NOX4, SOS2, RPL7L1, EIF3E, EIF4G1, CCND1, RPL15, PTBP1, GAB1, EIF3D, EIF4A1, SOS1, AKT3, FRS2, AGO4* |
| Myc Mediated Apoptosis Signaling | 2.30 | NA | *FADD, YWHAH, GAB1, SOS1, SOS2, MAPK9, AKT3, FRS2* |
| Cholecystokinin/Gastrin-mediated Signaling | 2.29 | 1.90 | *MAP2K6, SOS1, ITPR3, SOS2, MEF2A, MAPK9, PLCB1, MEF2C, ITPR1, BCAR1* |
| Regulation of eIF4 and p70S6K Signaling | 2.15 | 1.89 | *GAB1, EIF3D, PPM1J, EIF4A1, SOS1, SOS2, AKT3, EIF3E, EIF4G1, FRS2, PPP2R5A, AGO4, EIF4EBP1* |
| NRF2-mediated Oxidative Stress Response | 2.15 | 1.63 | *MAP2K6, DNAJC17, DNAJB12, DNAJB4, DNAJC6, MAP3K1, MAPK9, DNAJC13, DNAJC11, GAB1, CAT, CCT7, NFE2L2, DNAJC16, FRS2* |
| FGF Signaling | 2.14 | 3.00 | *MAP2K6, MET, GAB1, SOS1, MAP3K1, SOS2, AKT3, ITPR1, FRS2* |
| GDNF Family Ligand-Receptor Interactions | 2.09 | 1.34 | *GAB1, SOS1, ITPR3, SOS2, DOK7, MAPK9, ITPR1, FRS2* |
| Inhibition of Angiogenesis by TSP1 | 2.06 | NA | *SDC1, CD36, MAPK9, AKT3, GUCY1B3* |
| Synaptic Long Term Depression | 2.00 | 0.58 | *PNPLA8, PRKG1, NPR1, PPM1J, RYR2, ITPR3, GNA11, PLCB1, ITPR1, GNAZ, GUCY1B3, PPP2R5A* |

NA: z-score not available
